# DNA Nanostructure‐Templated Multivalency Enables Broad‐Spectrum Virus Inhibition

**DOI:** 10.1002/advs.202513710

**Published:** 2025-11-21

**Authors:** Saurabh Umrao, Abhisek Dwivedy, Dhanush Gandavadi, Chi Chen, Lifeng Zhou, Jinwei Duan, Vineetha Mareddy, Ying Fang, Xing Wang

**Affiliations:** ^1^ Department of Bioengineering University of Illinois at Urbana‐Champaign Urbana IL 61801 USA; ^2^ Nick Holonyak Jr. Micro and Nanotechnology Laboratory University of Illinois at Urbana‐Champaign Urbana IL 61801 USA; ^3^ Carl R. Woese Institute for Genomic Biology University of Illinois at Urbana‐Champaign Urbana IL 61801 USA; ^4^ Department of Pathobiology University of Illinois at Urbana‐Champaign Urbana IL 61801 USA; ^5^ Department of Advanced Manufacturing and Robotics Peking University Beijing 100871 China; ^6^ Department of Chemistry and Materials Science School of Sciences Chang'an University Xi'an Shaanxi 710064 China; ^7^ Department of Chemistry University of Illinois at Urbana‐Champaign Urbana IL 61801 USA; ^8^ Cancer Center at Illinois University of Illinois at Urbana‐Champaign Urbana IL 61801 USA

**Keywords:** broad‐spectrum antiviral, designer nanostructures, host–pathogen interactions, multivalency, programmable therapeutic nanomaterials, respiratory viruses, virus inhibition strategies

## Abstract

The rapid evolution and antigenic diversity of influenza A viruses (IAVs) continue to challenge antiviral strategies, highlighting the need for broadly effective and modular therapeutic platforms. While single‐domain nanobodies and DNA aptamer–based inhibitors have emerged as promising candidates, their efficacy is limited by monomeric binding to the hemagglutinin (HA) proteins populating the viral envelope. A programmable antiviral platform based on a honeycomb‐shaped designer DNA nanostructure (HC‐DDN) engineered to multivalently display HA‐targeting ligands with nanometer precision is presented. Two constructs are synthesized, HC–Nanobody and HC–Aptamer, organized in trimeric clusters to match the native HA trimer geometry. Using murine‐adapted H1N1 and H3N2 models, it is shown that both constructs outperform their free counterparts in viral neutralization and cytoprotection. HC–Nanobody construct achieves >99% inhibition of viral entry and improves cell viability by 35–45% at nanomolar concentrations. To assess translational relevance, the HC–Nanobody construct in a porcine IAV infection model is further evaluated, where it maintains high antiviral efficacy (>97% inhibition) and confers a 30–55% increase in cell viability relative to free nanobodies, confirming robust cross‐species performance. Overall, this work demonstrates the power of geometry‐matched multivalency to enhance viral neutralization and provides a rational blueprint for designing broad‐spectrum antivirals against rapidly evolving respiratory pathogens.

## Graphical abstract

A honeycomb‐shaped DNA nanostructure is introduced that organizes nanobodies or aptamers into trimeric clusters mirroring the native hemagglutinin arrangement on influenza viruses. This geometry‐matched multivalency enhances binding avidity by up to 1000‐fold, resulting in robust, broad‐spectrum viral neutralization and a 30–55% improvement in host cell viability in both murine and porcine infection models.

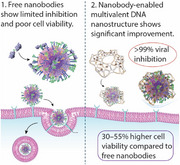

## 1 Introduction

Seasonal and pandemic outbreaks of influenza A virus (IAV) continue to pose a major global health challenge, driven by the virus's high mutation frequency and capacity for zoonotic reassortment.^[^ ^1^, ^2^, ^3^ ^]^ While vaccination remains the primary preventive strategy; current seasonal vaccines often provide suboptimal protection in vulnerable populations and can be undermined by antigenic drift or mismatches between vaccine strains and circulating lineages.^[^ ^4^, ^5^ ^]^ Alternative therapeutic options for IAV (e.g., neuraminidase inhibitors^[^ ^6^ ^]^ like oseltamivir, zanamivir, peramivir, and newer polymerase inhibitors^[^ ^7^ ^]^) are likewise limited by the rapid emergence of drug‐resistant variants^[^ ^8^ ^]^ and the narrow spectrum of strains, which has significantly reduced the clinical utility of frontline antivirals.^[^ ^9^ ^]^ Additionally, the FDA‐recommended cap‐dependent endonuclease inhibitor baloxavir marboxil, while effective at reducing symptom duration by ≈1 day, has prompted concerns due to the emergence of I38T/M/F polymerase mutations in ≈10% of treated patients, which compromise antiviral efficacy.^[^ ^10^ ^]^ There is an unmet clinical need for broad‐spectrum therapeutic agents against rapidly evolving influenza subtypes.

One promising strategy involves the development of broadly neutralizing antibodies (bnAbs) that target conserved epitopes within the influenza hemagglutinin (HA) surface glycoprotein, enabling cross‐reactivity across multiple IAV subtypes.^[^ ^11^, ^12^ ^]^ Several bnAbs such as CR6261,^[^ ^13^ ^]^ CR9114,^[^ ^14^ ^]^ and MEDI8852^[^ ^15^ ^]^ have demonstrated protective efficacy against diverse group 1 and group 2 HA subtypes in preclinical and human challenge studies.^[^ ^16^ ^]^ However, their application as routine prophylactic interventions is constrained by incomplete subtype coverage, as no single bnAb can neutralize the full diversity of circulating IAV strains.^[^ ^17^ ^]^ Moreover, practical limitations, including the need for high systemic doses, repeat administrations, and suboptimal biodistribution to the respiratory mucosa, pose significant challenges for sustained mucosal immunity. Large‐scale production costs, potential for antibody‐dependent enhancement,^[^ ^18^ ^]^ and the inherent large molecular size further complicate widespread deployment. These constraints have motivated us to look for innovative therapeutic platforms that combine the potent, strain‐transcending activity with enhanced tissue targeting, multivalency, and manufacturability to provide robust, affordable, and broadly effective antiviral protection.

Recent advances in biotherapeutics have highlighted the potential of nanobodies (single‐domain fragments derived from camelid heavy‐chain‐only antibodies) and aptamers (short nucleic acid ligands) as promising antiviral candidates due to their high specificity, stability, and ease of engineering.^[^ ^19^, ^20^ ^]^ Compared to conventional immunoglobulin G antibodies (≈150 kDa), nanobodies (≈15 kDa) and aptamers (≈5–15 kDa) are markedly smaller, and can be rapidly generated via immune library panning or SELEX (Systematic Evolution of Ligands by Exponential enrichment). Functionally, both nanobodies and aptamers have been shown to bind viral surface proteins‐such as the hemagglutinin (HA) of influenza A virus (IAV), with nanomolar to picomolar affinities, effectively inhibiting key steps in the viral life cycle, including receptor engagement and membrane fusion.^[^ ^19^, ^20^ ^]^ Their capacity to recognize structurally conserved or conformationally recessed regions, such as the HA stem domain, makes them particularly attractive for countering the continual antigenic drift and shift that characterize IAV evolution. However, when deployed in monovalent formats, these binders often lack sufficient avidity and fail to achieve broad neutralization across IAV subtypes.^[^ ^21^ ^]^ To address these limitations, Laursen et al. engineered synthetically linked multidomain nanobodies (MDNs), in which two or more nanobody units are fused via flexible peptide linkers to enable cooperative binding to multiple conserved epitopes.^[^ ^19^ ^]^ Notably, MDN constructs such as MD2407, which comprises four interlinked nanobodies (SD38–SD36–SD83–SD84), and MD3606 (MD2407 fused to a human IgG1 Fc domain) have demonstrated cross‐reactivity against both group 1 and group 2 IAVs, representing a significant leap toward universal nanobody‐based influenza therapeutics.^[^ ^19^ ^]^

Despite this progress, free‐floating MDNs face inherent biophysical constraints. While they offer enhanced functional valency, their conformational freedom and reliance on molecular diffusion limit their optimal engagement with the spatially ordered HA trimers (≈16 nm intertrimer HA distance) arranged on the IAV envelope.^[^ ^22^, ^23^ ^]^ Consequently, steric hindrance, entropic penalties from flexible linkers, and misalignment between nanobody domains and viral architecture collectively reduce the effective cooperative multivalent engagement that is theoretically achievable. This structural mismatch highlights a critical need for novel design strategies that impose precise spatial organization and rigidity to fully harness the multivalent binding potential of nanobody and aptamer‐based antivirals.

The principle of multivalency, whereby a single binder engages multiple identical viral epitopes simultaneously, is fundamental to both viral infection and its inhibition. Viruses like IAV use densely packed HA trimers to bind host cell receptors with high avidity, exploiting multivalent interactions for robust cell entry.^[^ ^22^ ^]^ To competitively block this, antiviral inhibitors benefit greatly from geometry‐matched multivalent architectures in which binding units are spatially arranged to mirror the antigen distribution on the viral surface. Recent advances in DNA nanotechnology have unlocked the ability to construct designer DNA nanostructures with sub‐nanometer spatial precision, enabling the controlled positioning of multiple ligands‐such as aptamers, peptides, or nanobodies—in defined geometric patterns.^[^ ^24^, ^25^, ^26^, ^27^, ^28^ ^]^ This ‘DNA‐based material organization’ provides a versatile toolkit for creating scaffolds that emulate the viral envelope's topography, thereby amplifying local ligand density and facilitating cooperative binding interactions that dispersed and untethered molecules cannot achieve.^[^ ^29^ ^]^ Moreover, DNA nanostructures exhibit good biocombability and biostability, which can be improved or tuned by surface coating,^[^ ^30^, ^31^, ^32^, ^33^ ^]^ UV crosslinking,^[^ ^34^ ^]^ or using aqueous ionic liquid solution for self‐assembly.^[^ ^35^ ^]^ These findings suggest the transformative potential of integrating DNA nanotechnology with next‐generation antiviral design to overcome the spatial and valency limitations inherent to free nanobodies and aptamers.

**Scheme 1:**
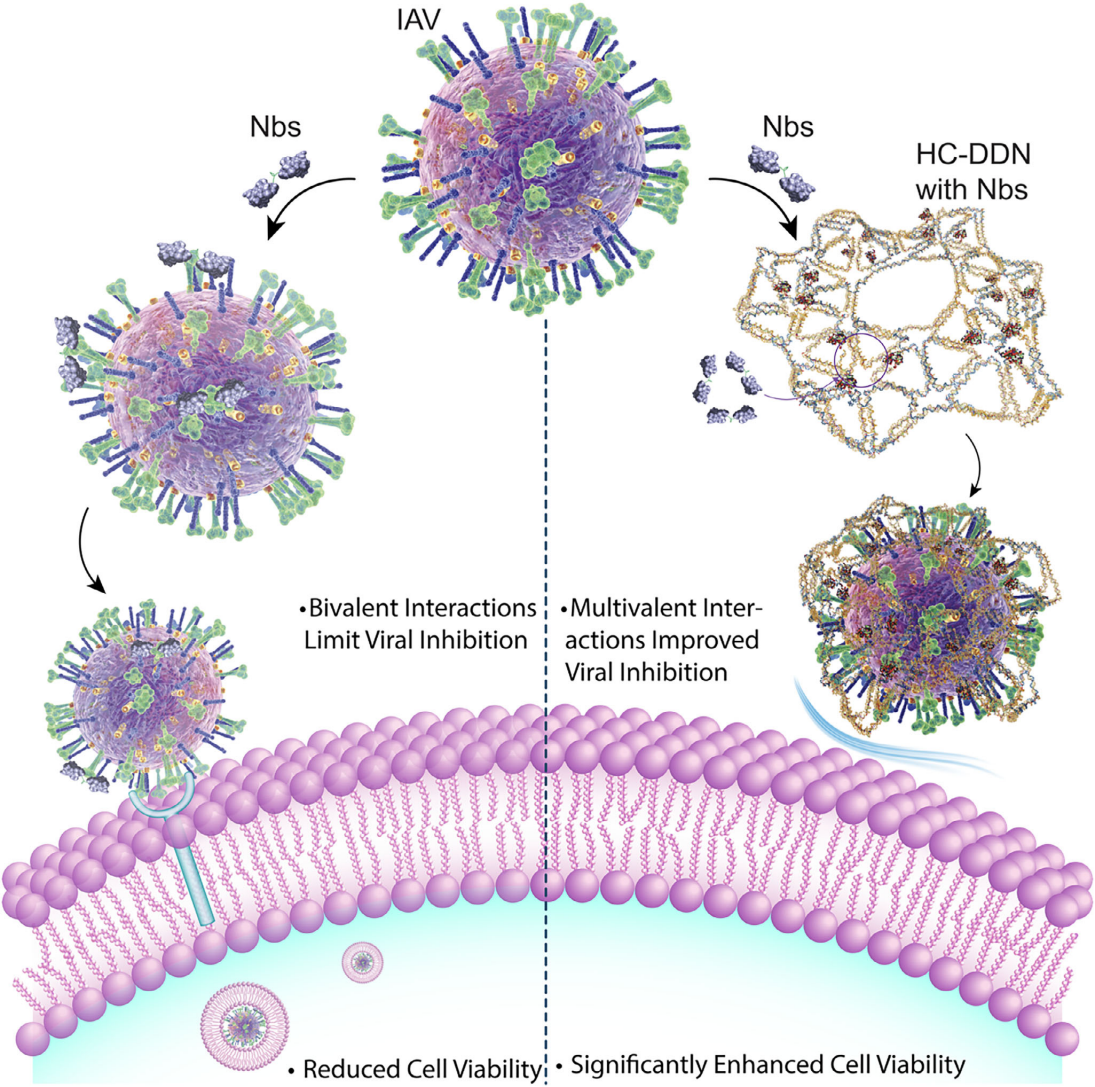
Schematic illustration of the honeycomb‐shaped designer DNA nanostructure (HC‐DDN)‐enabled antiviral platform. The HC‐DDN construct hosts antiviral agents, such as nanobodies (SD38–SD36, shown as an example), into trimeric patterns that promote multivalent interactions with hemagglutinin proteins on the influenza A virus (IAV) surface. This multivalent arrangement enables broad‐spectrum viral neutralization and significantly improves cell viability in both murine and porcine models.

In this study, we synthesized a honeycomb‐shaped designer DNA nanostructure^[^ ^36^ ^]^ (HC‐DDN) that spatially arranges antiviral ligands into trimeric patterns, providing the necessary bendability for the ligands to align with the native topology of influenza A virus (IAV) HA trimers (**Scheme** **1**). Previous multivalent antiviral strategies that include MATCH,^[^ ^37^ ^]^ DNA‐Star,^[^ ^24^ ^]^ and SNAP^[^ ^38^ ^]^ have been reported. While these studies share the conceptual framework of multivalent engagement to enhance neutralization, HC‐DDN is designed and utilized to address a distinct biophysical challenge and differs from prior strategies in scale, mechanism, and implementation (see Table S1, Supporting Information for detailed comparison). Specifically, we designed two classes of antiviral constructs: i) HC–Nanobody (or HC‐Nb) construct, in which synthetically fused dual‐domain nanobodies (SD38‐SD36)^[^ ^19^ ^]^ are arranged in a spatially matched trimeric format, and ii) HC–Aptamer (or HC‐Apt), which displays a previously characterized broad‐spectrum HA‐targeting DNA aptamer (UHA‐2)^[^ ^39^ ^]^ in a similar multivalent layout. We systematically characterized the binding performance of these constructs and found that patterned immobilization on the HC‐DDN platform enhanced binding affinity by ≈100–1000‐fold compared to their respective free forms. Consistent with this increased avidity, in vitro assays demonstrated corresponding improvements in antiviral efficacy. In murine‐adapted H1N1 and H3N2 models, both constructs exhibit enhanced viral inhibition and cytoprotection relative to monovalent controls. Specifically, the HC–Nb construct achieves between 95 and 99% viral inhibition and confers a 35–45% relative improvement in cell viability compared to an equivalent dose of free nanobody across tested IAV subtypes. Likewise, the HC–Apt construct provides ≈98% viral inhibition and a 15–45% relative increase in cell viability compared to free aptamer treatment. To further assess translational potential, we evaluated the constructs in a porcine‐adapted IAV infection model, which more closely replicates human influenza pathogenesis. In this physiologically relevant setting, the HC–Nb construct maintains robust antiviral efficacy and yields a 30–55% improvement in cell viability relative to free nanobody, demonstrating consistent cross‐species performance and resilience to subtype variations. Collectively, this work introduces a rationally designed multivalent scaffold for precise antiviral ligand patterning, thus maximizing binding avidity and functional neutralization through multivalent interactions. Our approach provides a versatile, modular blueprint for the development of advanced, broad‐spectrum antivirals not only against IAV but also for other rapidly evolving respiratory viruses of concern for One Health.^[^ ^40^ ^]^

## 2 Results and Discussion

### 2.1 DNA Nanostructure Design and Simulation Using oxDNA Tool

The planar honeycomb‐shaped designer DNA nanostructure (HC‐DDN) was adapted from a previous design^[^ ^36^, ^41^ ^]^ that used a wireframe double‐crossover motif combined with a dual‐graph scaffold routing strategy. This approach enables efficient routing of a single M13 DNA scaffold through complex geometric patterns, ensuring structural fidelity and minimizing topological strain. The final HC‐DDN consists of six interlinked hexagonal units, each with a minimum edge length of 42 base pairs, offering a modular and symmetrical platform suitable for functionalization of molecular binders (nanobody or aptamer). To improve the flexibility and structural robustness of the nanostructure, a discrete‐edge design was implemented, in which each edge junction was modified to include seven thymidine (T) bulges. These T‐bulges introduce localized flexibility at crossover points, allowing for smoother folding and reducing the risk of scaffold fraying or mismatched base pairing, which can otherwise compromise nanostructure integrity. The designed HC‐DDN structure was edited using oxView software,^[^ ^42^ ^]^ and three sets of single‐stranded DNA (ssDNA) overhangs, each with the sequence CGCAATAGCAGC, were appended in a trimeric configuration to each hexagonal unit. These ssDNA overhangs served as programmable anchoring points for the hybridization of either DNA–Nb conjugates or the UHA‐2 DNA aptamer via complementary base‐pairing (refer to the Methods section for detailed sequence list). The trimeric arrangement of each molecular binder (nanobody/aptamer) enables effective multivalent interactions with the densely packed HA trimers on the IAV envelope, thereby enabling high avidity that enhances viral inhibition efficiency.

**Figure 1:**
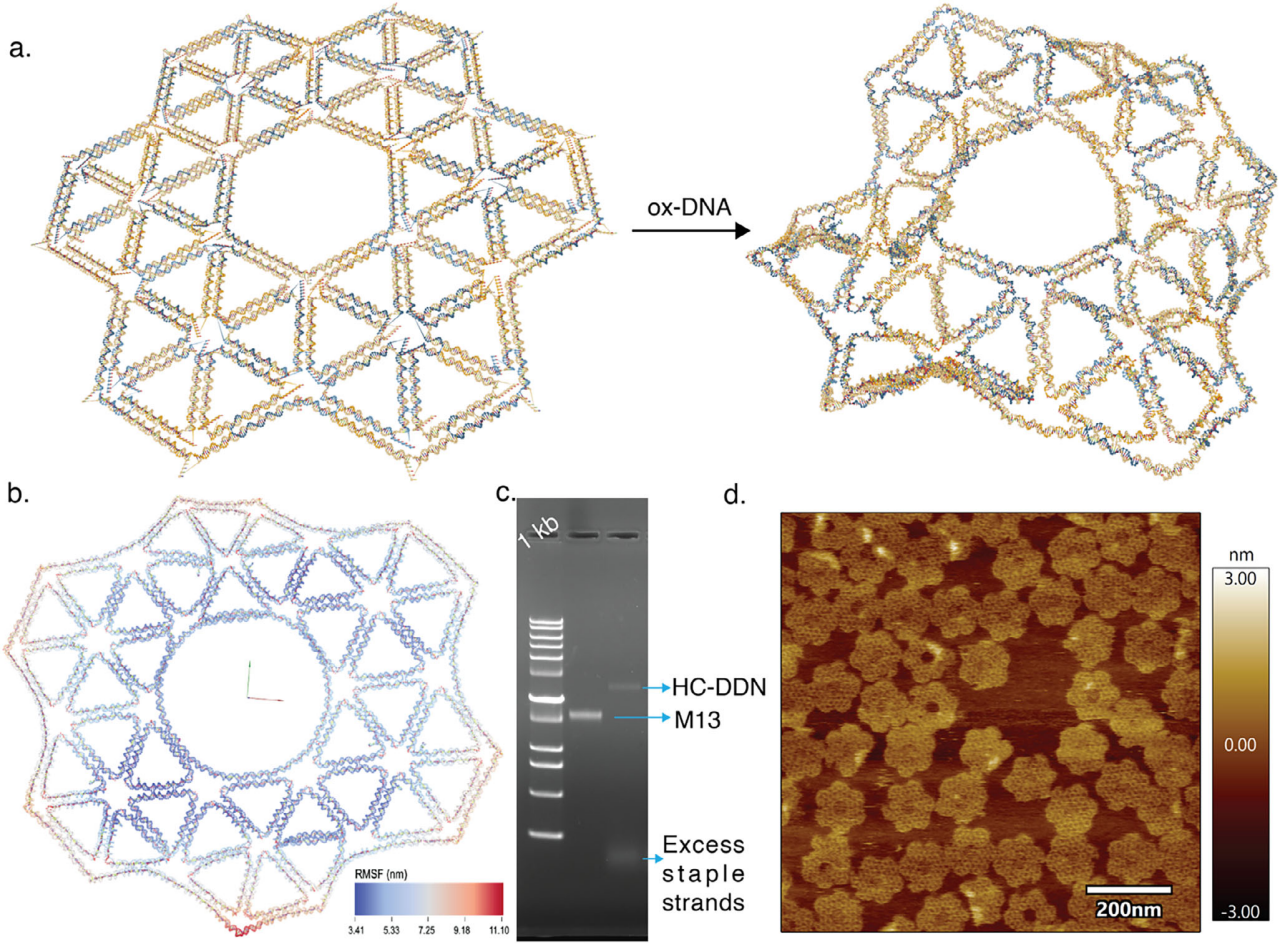
The oxDNA model, and the experimental validation of the honeycomb‐shaped DNA nanostructure (HC‐DDN). a) Structural behavior of the HC‐DDN was assessed using oxDNA simulations run for 10^9^ steps with an integration of 0.001 simulation time units. b) The resulting root mean square fluctuation (RMSF) map highlights regions of structural flexibility and stability, with RMSF values ranging from 3.41 nm (dark blue) to 11.10 nm (red). Dark blue regions indicate nucleotides with minimal fluctuations, while lighter blue to red denotes increasing dynamic motion of the structure. c) Successful assembly of the HC‐DDN was confirmed by 1% agarose gel electrophoresis (AGE). The assembled HC‐DDN structure showed slower migration relative to the M13 scaffold strand, suggesting the successful formation of the HC‐DDN construct. d) Atomic force microscopy (AFM) further provided nanoscale visualization, validating the expected morphology and correct folding of the HC‐DDN.

To evaluate the structural dynamics and stability of the HC‐DDN, we performed coarse‐grained molecular dynamics simulations using the oxDNA simulation engine (**Figure** **1**a; Movie S1, Supporting Information). We quantified structural flexibility by calculating the root mean square fluctuation (RMSF) for each nucleotide. RMSF values ranged from 3.41 nm to 11.10 nm (Figure 1b), with the central domains showing minimal fluctuation (dark blue), while peripheral regions and trimeric anchoring sites exhibit higher flexibility (shaded light blue to red). This distribution is consistent with the expected dynamic behavior of ssDNA overhangs and T‐bulged junctions. To assess structural stability, we monitored the potential energy of the system throughout the simulation. The energy decreases over time and plateaued at ≈–1.470 simulation energy units per particle (Figure S1a, Supporting Information), indicating convergence toward a stable, equilibrated configuration.^[^ ^43^ ^]^

We further analyzed the hydrogen bond occupancy parameter to evaluate base‐pairing stability over the simulation window. This metric quantifies the presence and persistence of hydrogen bonds, serving as an indicator of strand fraying or bond breaking, which may lead to structural destabilization. As shown in Figure S1b (Supporting Information), regions with strong base pairing appear in red (representing high H‐bond occupancy), whereas dynamic or unpaired regions are marked in blue (primarily the trimeric interfaces and T7 bulges). No significant white regions (partially bound states) were detected, suggesting a high degree of base‐pair retention throughout the structure. These computational findings indicate that the HC‐DDN nanostructure maintains overall structural integrity while allowing localized flexibility, an essential feature for its intended function as a multivalent antiviral scaffold.

### 2.2 DNA Nanostructure Synthesis and Characterization

To experimentally validate the successful assembly of the HC‐DDN construct, we employed 1% agarose gel electrophoresis (AGE) and atomic force microscopy (AFM) as complementary analytical techniques. AGE analysis confirms the formation of the desired nanostructure, with a distinct band shift observed in lane‐2 corresponding to the fully assembled HC‐DDN, as compared to the M13 DNA reference scaffold in lane‐1 (Figure 1c). The reduced electrophoretic mobility reflects the increased molecular weight and compact folding of the DNA nanostructure, indicating successful scaffold and staple strands hybridization. Further geometrical and morphological characterization by AFM confirms the formation of well‐defined, planar nanostructures with a high proportion of correctly folded assemblies (Figure 1d). The observed geometry is in excellent agreement with the intended in silico design, supporting the structural accuracy of the assembly process. This foundation enables reliable downstream functionalization with DNA–Nb conjugates or UHA‐2 DNA aptamers, facilitating the systematic investigation of multivalency effects on antiviral efficacy.

### 2.3 Physiochemical Stability of DNA Nanostructure

To assess stability under physiologically relevant conditions, HC‐DDN was incubated in 10% mouse serum at 37 °C and sampled over a 24 h time course (Figure S2, Supporting Information). AGE showed that HC‐DDN retained its structural integrity with minimal to no detectable degradation throughout the 24 h incubation, indicating resistance to serum‐mediated protein challenge. We also evaluated pH tolerance by incubating HC‐DDN in buffers spanning pH 3.0–9.0. AGE analysis demonstrated that the structure remained intact from pH 5.0–9.0; at pH 4.0 a modest mobility shift was observed (consistent with partial protonation and altered intermolecular interactions), and at pH 3.0, moderate degradation occurred, as expected for strongly acidic conditions (Figure S3, Supporting Information). These data indicate that HC‐DDN (and, by extension, HC–Nb and HC–Apt) remains stable across physiological serum and pH ranges.

### 2.4 DNA–Nb Conjugation and Attachment on HC‐DDN Platform

**Figure 2:**
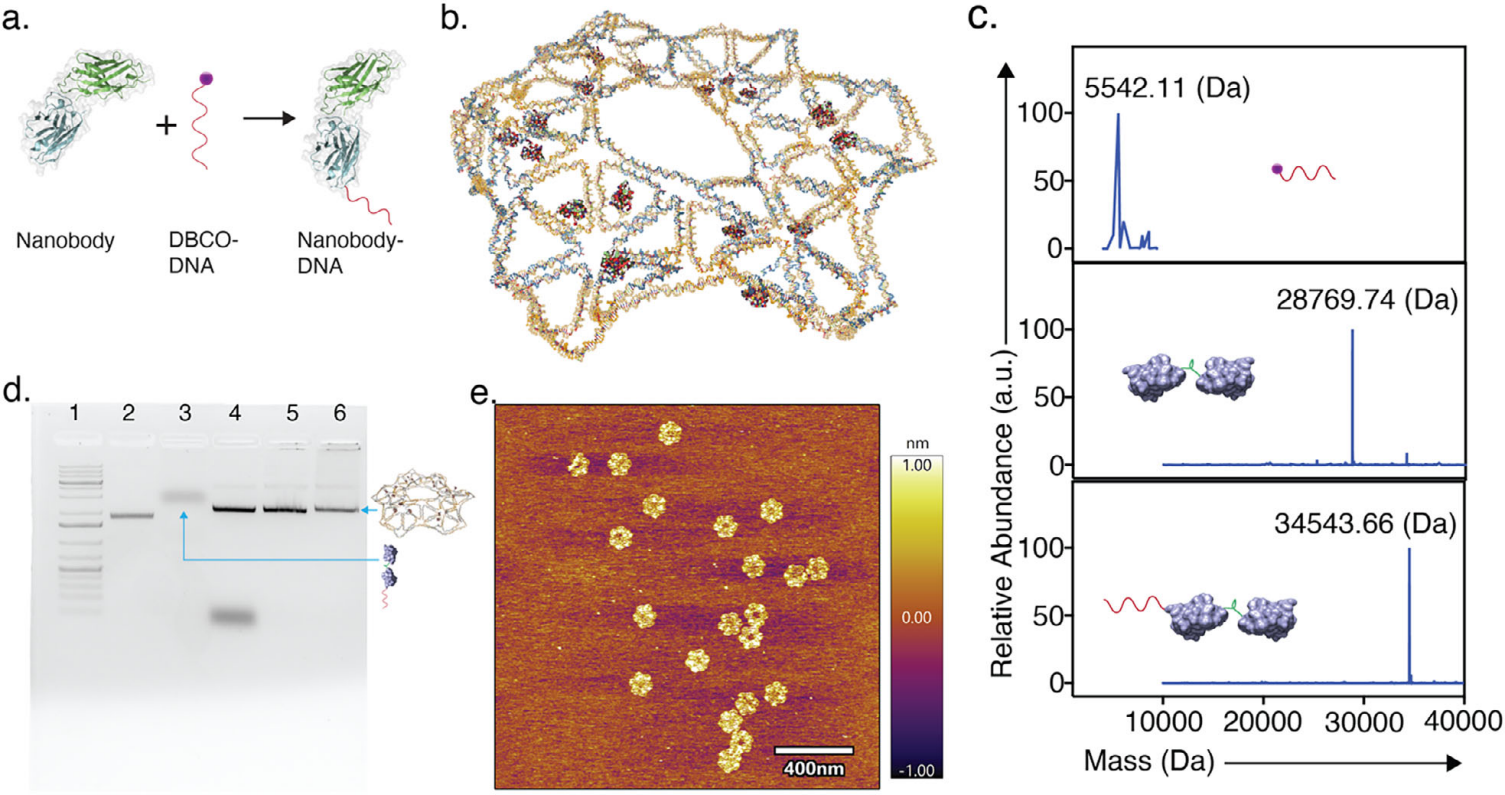
Synthesis, and characterization of DNA‐Nb construct. a) Schematic illustration of the site‐specific conjugation of a nanobody to a DBCO‐modified single‐stranded DNA (ssDNA). b) Schematic showing the precise attachment of DNA‐Nb conjugates onto the HC‐DDN, enabling spatially organized multivalent binding for enhanced viral inhibition. c) Native mass spectrometry analysis (Q Exactive UHMR hybrid Quadrupole‐Orbitrap, Thermofisher) verifying the masses of the DBCO‐ssDNA (top), free nanobody (middle), and the resulting ssDNA‐Nb conjugate (bottom). d) 1% agarose gel electrophoresis (AGE) validating the successful stepwise assembly of the HC–Nb construct. Lane 1: 1 kb plus DNA ladder; Lane 2: M13 DNA; Lane 3: DNA‐Nb conjugate; Lane 4: unpurified HC‐DDN origami; Lane 5: PEG‐purified HC‐DDN origami; Lane 6: HC‐DDN functionalized with DNA‐Nb conjugate. e) Atomic force microscopy (AFM) image showing localized white dots, indicating successful attachment of DNA–Nb conjugates to the HC‐DDN surface.

To covalently attach a ssDNA to a nanobody with high efficiency while preserving its antigen‐binding functionality, we adopted a site‐specific modification strategy adapted from established Gly–His tag azide labeling protocols.^[^ ^44^, ^45^, ^46^ ^]^ Briefly, the Gly–His tag located at the N‐terminus of the Nb is selectively acylated with 4‐methoxyphenyl 2‐azidoacetate, introducing a reactive azide moiety at N terminal. The azide‐modified Nb is then reacted with DBCO‐modified ssDNA via a click reaction to generate the DNA–Nb conjugate (**Figure** **2**a). These conjugates were designed to hybridize onto the HC‐DDN surface, yielding nanobody‐functionalized assemblies referred to as HC–Nb constructs (Figure 2b). To achieve this, we first characterized the DNA‐Nb conjugates using high‐resolution mass spectrometry (Figure 2c). The mass data confirm that conjugation of a ssDNA (MW: 5542.11 Da) increases the nanobody's molecular weight from 28 769.74 to 34 543.66 Da, precisely matching the expected mass for a successful 1:1 conjugation. These results demonstrate that the two‐step, site‐selective conjugation approach yields high‐purity DNA–Nb constructs, which were subsequently used to synthesize the HC–Nb construct.

We next verified the successful assembly of the HC–Nb construct using the AGE technique (Figure 2d). The nanobody carries a net positive charge (+1.44 at pH 7), which can influence the electrophoretic migration of the DNA–Nb conjugate (lane 3). Consistent with this, the DNA–Nb conjugate showed reduced mobility compared to negatively‐charged M13 (lane 2), unpurified HC‐DDN (lane 4), or purified HC‐DDN (lane 5), due to the combined effects of its increased molecular weight and the reduced overall negative charge. Upon hybridization of the DNA–Nb conjugate to the purified HC‐DDN (lane 6), the overall net negative charge density of the HC‐DDN complex is further reduced, resulting in slower migration relative to unmodified HC‐DDN. However, the HC–Nb construct (lane 6) still migrates faster than the free DNA–Nb conjugate (lane 3), because the entire construct retains a higher overall negative charge attributed to the highly negative charge carried by HC‐DDN. We finally confirmed the successful formation of HC‐Nb construct via AFM imaging, which revealed localized white dots indicative of DNA–Nb presence on the HC‐DDN surface (Figure 2e). We mapped the height distribution along each edge of the honeycomb vertices and extracted profiles at regions where Nbs are expected to be displayed (Figure S4, Supporting Information). These profiles were then directly compared with the corresponding regions of the HC‐DDN scaffold without Nb conjugation. The measured height distribution of HC‐Nb constructs (1.430 ± 0.174 nm) was greater than that of HC‐DDN control (0.613 ± 0.040 nm), consistent with the expected increase due to Nb conjugation. Together, these results confirm both the successful synthesis of DNA–Nb conjugates and their site‐specific integration into the HC‐DDN surface, establishing a robust platform for downstream multivalent antiviral applications.

To evaluate whether the geometrically patterned 3Nb construct confers strong binding to the HA trimer, we performed computational structural analysis. The SD38–SD36 dual‐domain nanobody was first modeled ab initio using the Robetta server^[^ ^47^ ^]^ and subsequently docked onto the HA monomer with the HADDOCK webserver^[^ ^48^ ^]^ (Figure S5a, Supporting Information). The top‐ranked binding mode, in which the dual nanobody engages previously reported HA residues,^[^ ^19^ ^]^ was used to generate three structural models: HA trimer bound to a single nanobody, two nanobodies, or three nanobodies arranged in the 3Nb configuration. The three‐nanobody–HA complex was designed to mimic the geometric pattern of the HC–Nb construct. We performed energy minimization, which revealed that the 3Nb–HA complex exhibited the most favorable binding free energy (Figure S5b, Supporting Information), surpassing even the native HA–sialic acid interaction.

Furthermore, we also examined the nanobody–HA interaction interface and found the key contact residues to be conserved across both murine‐ and porcine‐adapted H1N1, as well as the H3N2 subtype. Briefly, we performed multiple sequence alignment analysis (MSA) of HA proteins from the mouse‐ and pig‐adapted H1N1 and H3N2 strains. The residues predicted to interact with the SD38–SD36 dual nanobody construct (e.g., G23, V36, N40, T44, L49, L50, L58, I282, T316; numbering according to pig H1N1 HA) were found to be either strictly conserved or substituted with chemically similar amino acids (e.g., V→I, L→I, N→Q) (Figure S6, Supporting Information). This high level of conservation strongly suggests that the binding interface of the dual nanobody is preserved across all four viral strains. Taken together, these observations suggest that the geometrically patterned three‐nanobody design mimicking the HC‐Nb construct may provide strong and stable interactions with the HA trimer irrespective of viral host or subtypes.

### 2.5 Quantification of Binding Affinity for Free Nanobodies and the HC–Nb Construct Using Surface Plasmon Resonance (SPR) method

**Figure 3:**
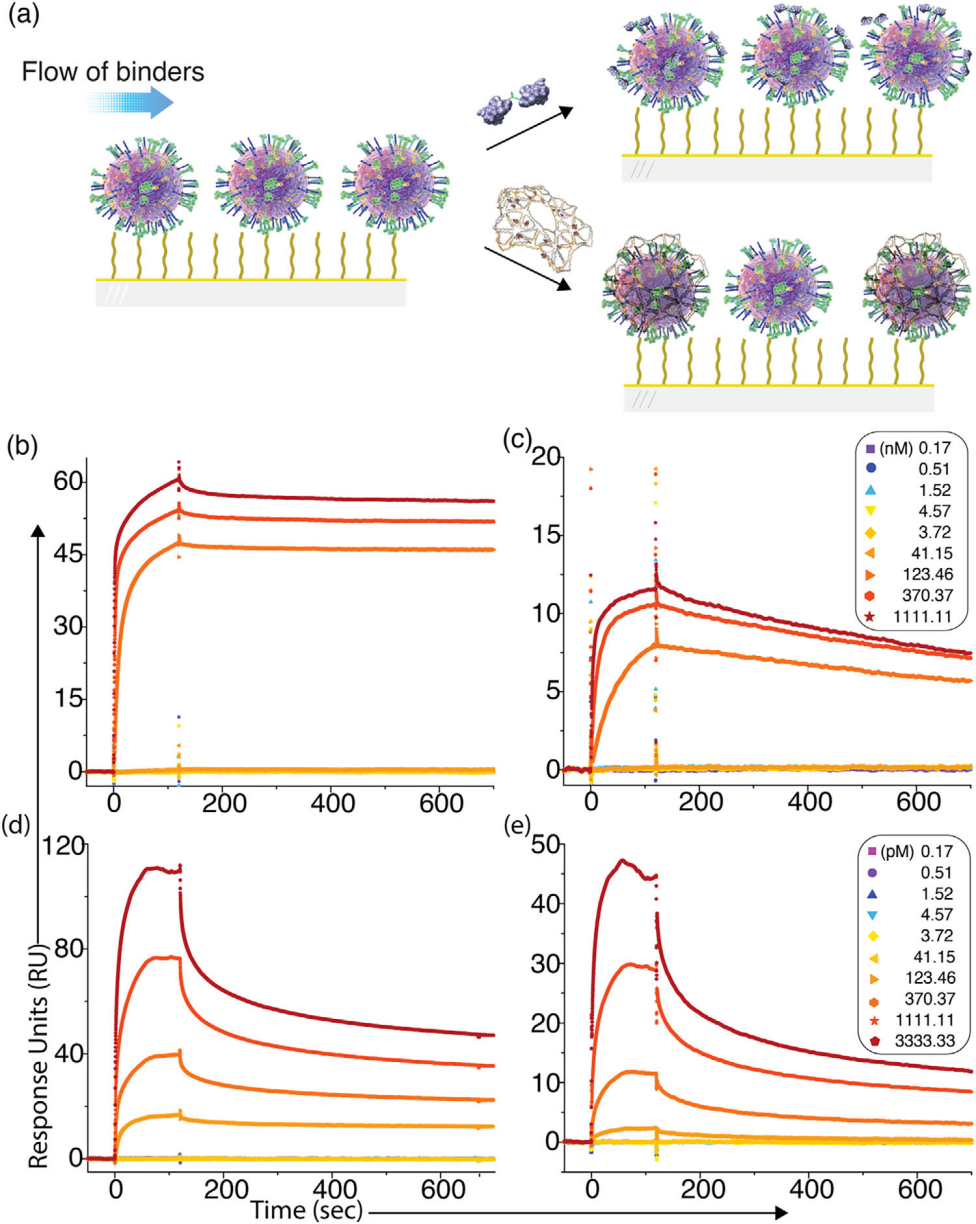
Surface plasmon resonance (SPR) assay for measuring binding affinities of antiviral inhibitors. a) Schematic of the SPR experimental workflow: whole H1N1 and H3N2 virions (≈1 × 10^6^ copies per mL, diluted in 10 mm sodium acetate buffer, pH 5.0) were immobilized on separate flow cells of a CM5 sensor chip via standard amine coupling. Nanobodies or HC–Nb constructs were injected over the immobilized virions for an association phase of 120 s, followed by a 600 s dissociation phase. b,c) Representative SPR sensorgrams showing the binding interactions of free nanobodies with immobilized H1N1 b) and H3N2 c) virions at concentrations ranging from 0.17 nm to 1.11 µm. d,e) SPR sensorgrams showing the binding interactions of HC–Nb constructs with immobilized H1N1 d) and H3N2 e) virions at concentrations ranging from 0.17 pm to 3.33 nm.

We employed the surface plasmon resonance (SPR) method to quantitatively assess how multivalent presentation on a DNA nanostructure influences nanobody binding to HA proteins on the IAV surface. For this purpose, inactivated H1N1 and H3N2 virions were immobilized onto separate flow cells of a CM5 sensor chip via standard amine coupling (**Figure** **3**a).^[^ ^49^ ^]^ Free nanobodies were injected at concentrations ranging from 0.17 nm to 1.11 µm to determine their binding affinities to the HA epitopes displayed on intact virions (Figure 3b,c).^[^ ^50^ ^]^ We found that the free nanobodies demonstrate strong binding avidity with equilibrium dissociation constants (*K_D_*) of 4.58 nm for H1N1 (Figure 3b), and 10.80 nm for H3N2 (Figure 3c). Next, to evaluate whether site‐specific conjugation influences nanobody performance, we measured the binding affinity of the ssDNA–Nb against immobilized H1N1, which shows a comparable *K_D_* of 3.83 nm, indicating that N‐terminal ssDNA attachment does not interfere with the nanobody's antigen recognition ability (Figure S7, Supporting Information). Furthermore, we assessed the specificity of the nanobody by injecting varying concentrations of ssDNA‐Nb conjugate (0.17 nm–1.11 µm) over a separate flow cell immobilized with SARS‐CoV‐2 virus as a non‐target control. The SPR sensorgrams show negligible binding of the nanobody to SARS‐CoV‐2, which displays a trimeric spike protein arrangement similar to the HA distribution on IAV (Figure S8, Supporting Information). This result confirms the high target selectivity of the DNA–Nb for influenza HA epitopes.

We then assembled multiple ssDNA–Nb units onto the HC‐DDN platform to generate a multivalent nanobody construct, HC–Nb. This construct was injected at concentrations ranging from 0.17 pm to 3.33 nm over the same sensor chip containing immobilized H1N1 and H3N2 virions. The multivalent configuration of HC–Nb produces a striking enhancement in binding affinity, with *K_D_* values of 0.35 pm for H1N1 (Figure 3d), and 6.69 pm for H3N2 (Figure 3e), representing an improvement of approximately three log folds compared to the free nanobody or previously reported MDNs.^[^ ^19^ ^]^ The substantial avidity boost is attributed to cooperative multivalent interactions enabled by the spatially organized display of multiple nanobodies on the HC‐DDN scaffold, which facilitates simultaneous engagement of multiple HA epitopes, a feature unattainable with monomeric nanobodies alone. Overall, this pronounced increase in binding avidity translated into greatly enhanced antiviral efficacy demonstrated by the in vitro assays (see flow cytometry results in later sections), suggesting the functional advantage of the pattern recognition–enabled multivalent strategy.

### 2.6 Enhanced Aptamer Affinity via DNA Nanostructure Presentation

Aiming to test whether multivalent presentation on a virion‐scale DNA scaffold could enhance apparent binding across different binder classes, we further explored DNA aptamers as an alternative to Nbs. To this end, we selected two well‐characterized HA‐binding DNA aptamers (UHA‐2^[^ ^36^ ^]^ and V46^[^ ^51^ ^]^) with distinct epitope specificities. UHA‐2 DNA aptamer targets the HA head and has been reported to exhibit inhibitory activity against influenza A virus, making it an appropriate candidate for assessing whether geometry‐matched multivalent display on HC‐DDN could enhance neutralization potency. By contrast, V46 DNA aptamer binds the conserved HA stem but has not been reported to possess intrinsic inhibitory activity. Therefore, V46 is used as a mechanistic reporter to test whether multivalent display increases apparent binding at a nonblocking epitope. Together, these aptamers enabled us to probe both the generality of affinity enhancement by HC‐DDN and the extent to which epitope location dictates if binding gains could translate into improved antiviral function.

First, to validate multivalent aptamer display on HC‐DDN, we employed an AGE‐based loading assay. HC‐DDN, which contains 18 designated binding sites, was annealed with FAM‐labeled V46 aptamers at scaffold‐to‐aptamer ratios ranging from 1:0 to 1:36. AGE analysis revealed a progressively increased FAM fluorescence signal of the HC‐DDN band with increasing aptamer input, along with the appearance of free excess aptamer at higher ratios (Figure S9, Supporting Information). These results confirm efficient aptamer incorporation into the docking sites displayed on HC‐DDN. Next, by following a similar experimental approach, we injected varying concentrations of free UHA‐2 aptamer (0.17 nm–10 µm) over the flow cells containing immobilized H1N1, and H3N2 virions (Figure S10a,b, Supporting Information). Consistent with its reported broad‐spectrum binding, the free UHA‐2 aptamer displays strong affinity for both subtypes, with *K_D_* values of 19.09 nm for H1N1 (Figure S10a, Supporting Information), and 2.32 nm for H3N2 (Figure S10b, Supporting Information), which aligns well with prior reports demonstrating low‐to‐moderate nanomolar binding to various HA subtypes.

To assess whether multivalent presentation could similarly enhance aptamer binding, we constructed the HC–Apt platform, in which multiple copies of the UHA‐2 aptamer were site‐specifically anchored onto the trimeric protrusions of the HC‐DDN scaffold. The spatial arrangement is designed to mimic the native trimeric distribution of HA spikes on the viral envelope, thereby promoting simultaneous engagement of multiple HA binding sites. Following a similar methodology, we injected the HC‐Apt construct at concentrations ranging from 3 pm to 20 nm over both flow cells (Figure S10c,d, Supporting Information). SPR analysis revealed that this multivalent configuration greatly increases the apparent binding affinity by roughly an order of magnitude, improving the *K_D_* values to 0.10 nm for H1N1 (Figure S10c, Supporting Information), and 0.31 nm for H3N2 (Figure S10d, Supporting Information).

Since we aimed to employ both the HC–Nb and HC–Apt constructs in in vitro flow cytometry–based experiments for quantitative evaluation of antiviral efficacy, we incorporated the fluorescence (FAM)‐labeled DNA aptamer V46 as a signaling marker. The V46 aptamer was originally selected for its ability to bind the conserved stem region of the HA protein, enabling recognition of multiple IAV subtypes.^[^ ^52^ ^]^ However, as it has not been reported to possess inhibitory activity, we utilized V46 DNA aptamer as a reporter in our cell‐based assays. Following the same SPR protocol used for UHA‐2, we first characterized the binding affinity of the free V46 aptamer against immobilized H1N1 and H3N2 virions (Figure S11, Supporting Information). The free V46 aptamer demonstrated *K_D_* values of 7.88 nm for H1N1 (Figure S11a, Supporting Information) and 22.19 nm for H3N2 (Figure S11b, Supporting Information), in agreement with the previous report. Next, using the same assembly approach as for the HC–Apt construct, we functionalized the HC‐DDN platform with V46 to generate the HC–V46 construct. This multivalent configuration dramatically improves apparent binding affinity, lowering the *K_D_* value to ≈2.72 pm for H1N1 (Figure S11c, Supporting Information) and 87.92 pm for H3N2 (Figure S11d, Supporting Information), representing an enhancement of nearly three log folds compared to the free aptamer. The resulting FAM‐labeled HC–V46 construct was subsequently used to monitor viral entry and infection in all cell‐based assays reported in this study

### 2.7 HC–Nb Constructs Provide Robust Inhibition of Influenza A Virus Infections Across Murine Models

To quantify the antiviral efficacy of the HC–Nb construct in reducing IAV infection in Madin–Darby Canine Kidney (MDCK) cells, we employed our previously reported flow cytometry–based assay,^[^ ^26^ ^]^ enabling precise quantification of infected cell population by measuring the expression of a FAM‐labeled HC‐V46 construct. As a non‐binding negative control, we used the HC–Ova construct, in which an ssDNA‐modified ovalbumin (Ova) peptide was anchored onto the HC‐DDN using the same methodology as for HC–Nb and HC–Apt.

**Figure 4:**
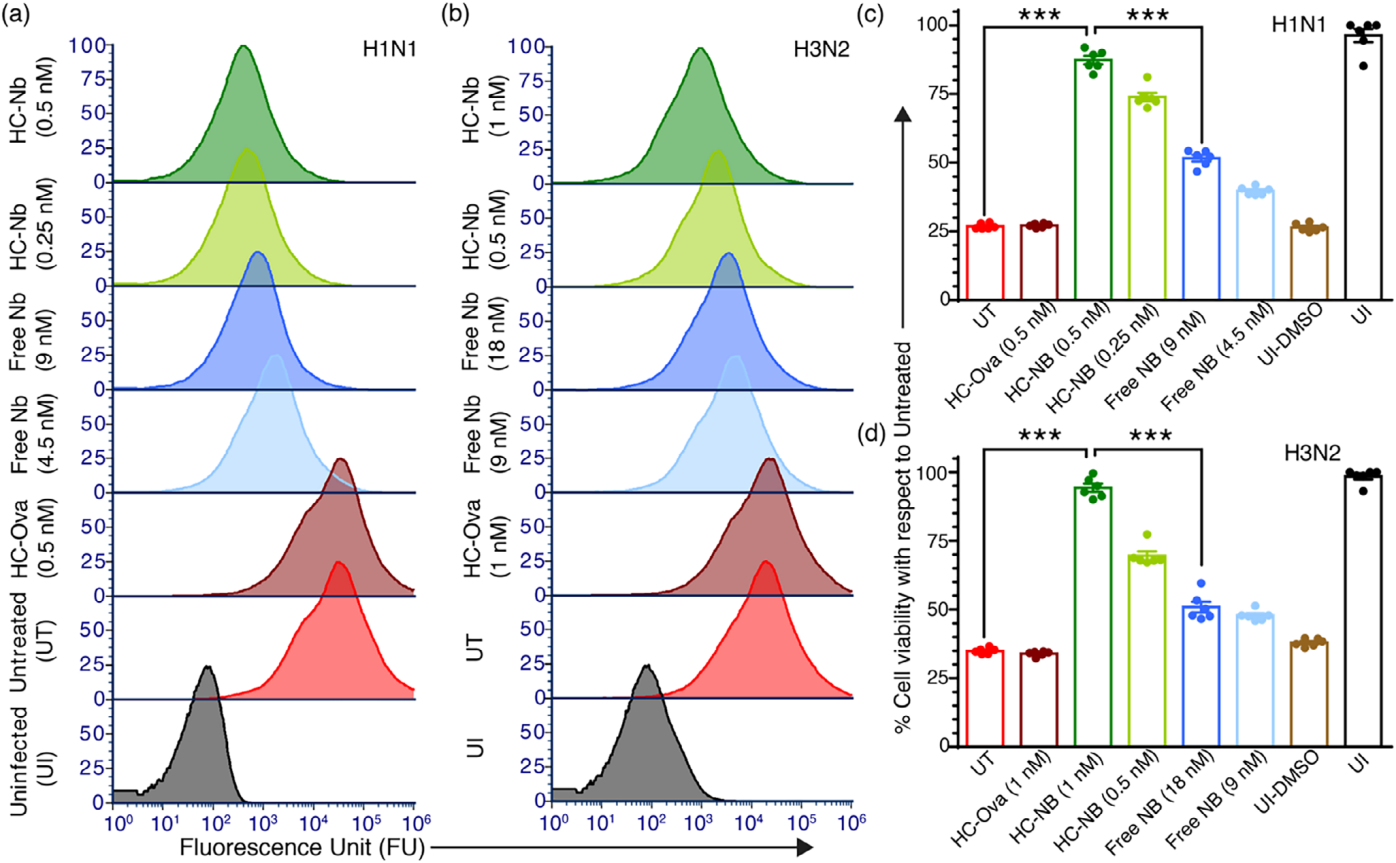
HC–Nb construct improves antiviral activity and limits cellular entry of murine‐adapted influenza viruses. a) Flow cytometry performed 1‐h postinfection shows that the HC–Nb construct more effectively blocks the entry of murine‐adapted H1N1 virions into host cells compared to free nanobodies. Controls include the HC scaffold hosting a non‐specific peptide (ovalbumin, Ova), untreated virions, and uninfected cells. b) A similar but more pronounced improvement is observed for H3N2 virions, highlighting the construct's enhanced neutralization capacity against this subtype. c,d) MTT assays conducted 24 h postinfection demonstrate that treatment with the HC–Nb construct significantly improves cell viability for H1N1 c) and H3N2 d) compared to free nanobodies, indicating effective viral suppression and cellular protection (DMSO‐treated uninfected cells serve as a positive control for cell death; bars represent mean and standard error; ^***^ represents a *p*‐value < 0.0001).

Prior to initiating infection assays, murine‐adapted IAV subtypes (H1N1 and H3N2) were pretreated either with free nanobody at their reported half‐maximal inhibitory concentrations (IC_50_; 9 nm for H1N1 and 18 nm for H3N2) or with the HC–Nb construct at concentrations adjusted to deliver an equivalent total nanobody content (0.5 nm for H1N1 and 1 nm for H3N2, considering 18 nanobody molecules per construct by design). This strategy allowed for a direct comparison of antiviral efficacy between monovalent and multivalent formats under equivalent binding stoichiometry. The infection assays were initiated by exposing MDCK cells to a high multiplicity of infection (MOI = 1000) of the respective IAV subtypes, following a 2‐h pre‐incubation of the virus with either free nanobody or HC–Nb at 37 °C. These temperatures and incubation period were selected to closely replicate physiological conditions, as IAV binding and internalization typically occur within the first 15 min and continue for up to an hour.^[^ ^27^ ^]^ Flow cytometry performed 1‐h postinfection reveals that the HC–Nb construct can greatly reduce infection levels compared to the free nanobody for both viral subtypes (**Figure** **4**a,b). More specifically, in the H1N1 model, treatment with 0.5 nm HC–Nb resulted in a mean fluorescence intensity of ≈200 RFU, approaching the uninfected baseline (≈20 RFU), suggesting >99% inhibition (Figure 4a; Figure S12a, Supporting Information). In contrast, treatment with 9 nm free nanobody yielded ≈500 RFU, corresponding to ≈97.5% inhibition. Similarly, in the H3N2 model, 1 nm HC–Nb achieved ≈500 RFU, whereas 18 nm free nanobody resulted in ≈2000 RFU, corresponding to 95% and 80% inhibition, respectively (Figure 4b; Figure S12b, Supporting Information). The extent of inhibition by the HC–Nb construct was particularly pronounced in the H3N2 model.

To assess whether the observed viral inhibition improvement could also confer cellular protection, we performed MTT assays to measure cell viability post‐infection. In the H1N1 model, untreated or HC–Ova–treated cells exhibit ≈25% loss in viability due to virus‐induced cytopathic effects. In contrast, cells infected with virions that were pretreated with 0.25 and 0.5 nm HC–Nb retained >75% and >85% viability, respectively—comparable to uninfected controls (Figure 4c). Free nanobody at equivalent nanobody content (4.5 and 9 nm) provides only modest protection (40–50% viability), highlighting the inherent limitations of free nanobodies relative to the multivalent HC–Nb construct. In the H3N2 model, the HC–Nb construct again demonstrates superior protection. At 1 nm, HC–Nb restores cell viability to ≈95%, while free nanobody at 18 nm achieves only ≈50% viability (Figure 4d).

Interestingly, while the flow cytometry–based characterization of viral inhibition shows only a modest improvement for the HC–Nb construct compared to the free nanobody (e.g., >99% vs ≈97.5% inhibition for H1N1, and 95% vs 80% inhibition for H3N2), the corresponding impact on cell viability was substantially greater (a relative difference of ≈35% for H1N1 and ≈45% for H3N2). This apparent discrepancy can be explained by the exponential nature of viral replication, wherein even a small residual population of infectious virions that escapes neutralization can amplify within host cells, propagate to neighboring cells, and induce significant cytopathic effects.^[^ ^53^, ^54^, ^55^ ^]^ We postulate that the HC–Nb construct's multivalent architecture not only improves initial binding avidity but also enhances the durability and robustness of HA trimer engagement throughout the infection window. Thus, it can suppress viral propagation more effectively within the cell monolayer. Consequently, this translates into greatly improved cell viability despite the relatively small difference in early inhibition assays. Overall, these findings demonstrate that multivalent nanobody‐functionalized DNA nanostructures significantly enhance antiviral efficacy and cytoprotection under physiologically relevant conditions.

### 2.8 Multivalent HC–Apt Constructs Enhance Antiviral Efficacy Against IAV Subtypes in Murine Models

Following the same cell infection and flow cytometry–based methodology used for the HC–Nb construct, we evaluated the antiviral performance of both the free UHA‐2 aptamer and the multivalent aptamer‐functionalized DNA nanostructure (HC–Apt) to demonstrate the versatility of our platform. Compared to nanobodies, the reported half‐maximal inhibitory concentrations (IC_50_) for the UHA‐2 aptamer are considerably higher, estimated at ≈378 nm for H1N1 and 470 nm for H3N2. Accordingly, we adjusted the HC–Apt dose in subsequent experiments to match the equivalent total aptamer content for an appropriate comparison. As a negative control, we employed a previously reported aptamer specific to the receptor‐binding domain (RBD) of the SARS‐CoV‐2 spike glycoprotein,^[^ ^56^ ^]^ which was anchored to the HC‐DDN platform using the same assembly protocol as for HC–Apt, resulting in the HC–RBD construct.

**Figure 5:**
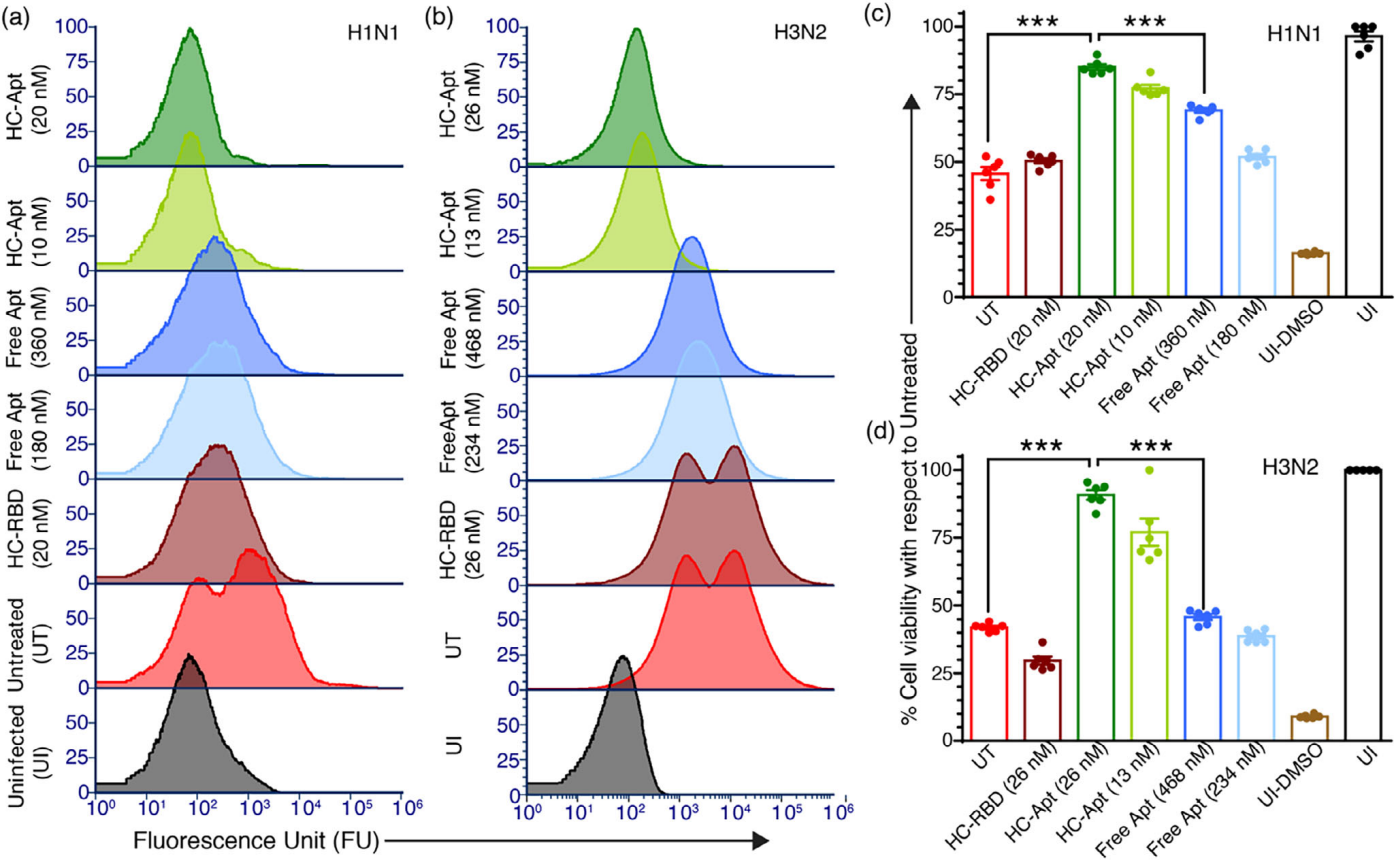
HC–Aptamer construct boosts antiviral performance and restricts host cell invasion by murine‐adapted influenza viruses. a) Flow cytometric analysis at 1‐h postinfection indicates that the HC–Apt construct more efficiently inhibits H1N1 virion entry into host cells than free aptamers. Controls include the HC scaffold functionalized with a nontargeting aptamer (specific to the SARS‐CoV‐2 spike RBD), untreated virions, and uninfected cells. b) A similar trend is observed for H3N2 virions, with a more pronounced antiviral effect relative to free aptamer treatment. c,d) MTT assays 24 h after infection show that HC–Apt treatment significantly enhances cell viability for H1N1 c) and H3N2 d) infections compared to free aptamers, suggesting lower viral replication and better cellular protection (DMSO‐treated uninfected cells serve as a positive control for cell death; bars indicate mean and standard error; ^***^ *p* < 0.0001).

Flow cytometry analysis reveals that the HC–Apt construct displays markedly improved antiviral efficacy compared to the free UHA‐2 aptamer against both H1N1 and H3N2 IAV subtypes in murine‐adapted models (**Figure** **5**a,b). In the H1N1 model, treatment with 20 nm HC–Apt reduces the mean relative fluorescence intensity to ≈120 RFU—only slightly above the uninfected control (≈45 RFU), indicating >98% inhibition of viral entry (Figure 5a; Figure S13a, Supporting Information). In contrast, treatment with 360 nm free aptamer (approximating its EC_50_) produces a much higher fluorescence signal (≈2000 RFU), corresponding to only ≈75% inhibition. A similar trend has been observed in the H3N2 model, where treatment with 26 nm HC–Apt reduces the fluorescence intensity to ≈150 RFU (≈98% inhibition), while administration of 468 nm free aptamer (near its EC_50_) results in a much higher signal of ≈1800 RFU (≈74% inhibition) (Figure 5b; Figure S13b, Supporting Information). To determine whether this enhanced antiviral effect could translate into improved cellular protection, we conducted MTT assays using H1N1‐infected MDCK cells treated with HC–Apt, free aptamer, or the nonbinding control HC–RBD. Cells exposed to untreated virus or virus pre‐incubated with HC–RBD show a great reduction in viability (≈50%), consistent with virus‐induced cytopathic effects and the ineffectiveness of the control HC‐RBD construct (Figure 5c). In contrast, cells treated with 10 and 20 nm HC–Apt exhibit viability of ≈78% and ≈85%, respectively, comparable to uninfected controls, indicating robust cytoprotection. By comparison, free aptamer treatments at 180 and 360 nm (equivalent to aptamer content in the HC–Apt doses) offered limited protection, restoring viability to only ≈50% and ≈70%, respectively. These results indicate that free aptamers, despite being present at high concentrations, lack the spatial arrangement and cooperative binding necessary to effectively block viral entry and protect host cells. Compared to H1N1 infection model, we observed a similar but more pronounced effect in the H3N2 infection model. Specifically, treatment with 26 nm HC–Apt restores cell viability to ≈90%, whereas the same aptamer delivered in free form at 468 nm fails to confer significant protection (≈45% viability), comparable to the negative control (Figure 5d). These results further support the advantage of DNA nanostructure‐based multivalent aptamer organization in maximizing therapeutic benefit through improved viral neutralization and cellular protection.

Direct comparison of the HC–Apt and HC–Nb constructs across murine IAV models reveals that while both formats could enhance antiviral efficacy, the HC–Nb consistently achieves superior cytoprotection. This observation aligns with previous reports,^[^ ^53^, ^54^ ^]^which demonstrate that nanobodies generally outperform aptamers as viral inhibitors due to their high intrinsic affinity and capacity to neutralize conformational epitopes inaccessible to most synthetic ligands. When integrated into the HC‐DDN platform, the inherent potency of nanobodies is further amplified through multivalent spatial presentation, enabling near‐complete viral neutralization at substantially low molar concentrations.

### 2.9 HC–Nb Constructs Maintain Potent Antiviral Activity in Porcine Models

Encouraged by these promising findings, we extended our evaluation of the HC–Nb construct to a porcine model, which more closely recapitulates human influenza pathogenesis. Unlike mice, pigs are natural intermediate hosts for IAVs and develop clinical symptoms such as fever, nasal discharge, and respiratory distress that closely mirror human disease. These physiological and immunological similarities make pigs a robust and translationally relevant model for assessing the efficacy of antiviral interventions and therapeutic candidates.^[^ ^55^ ^]^ Therefore, testing the HC–Nb construct against porcine‐adapted IAV strains provides a more relevant framework for gauging its therapeutic potential in human clinical contexts.

**Figure 6:**
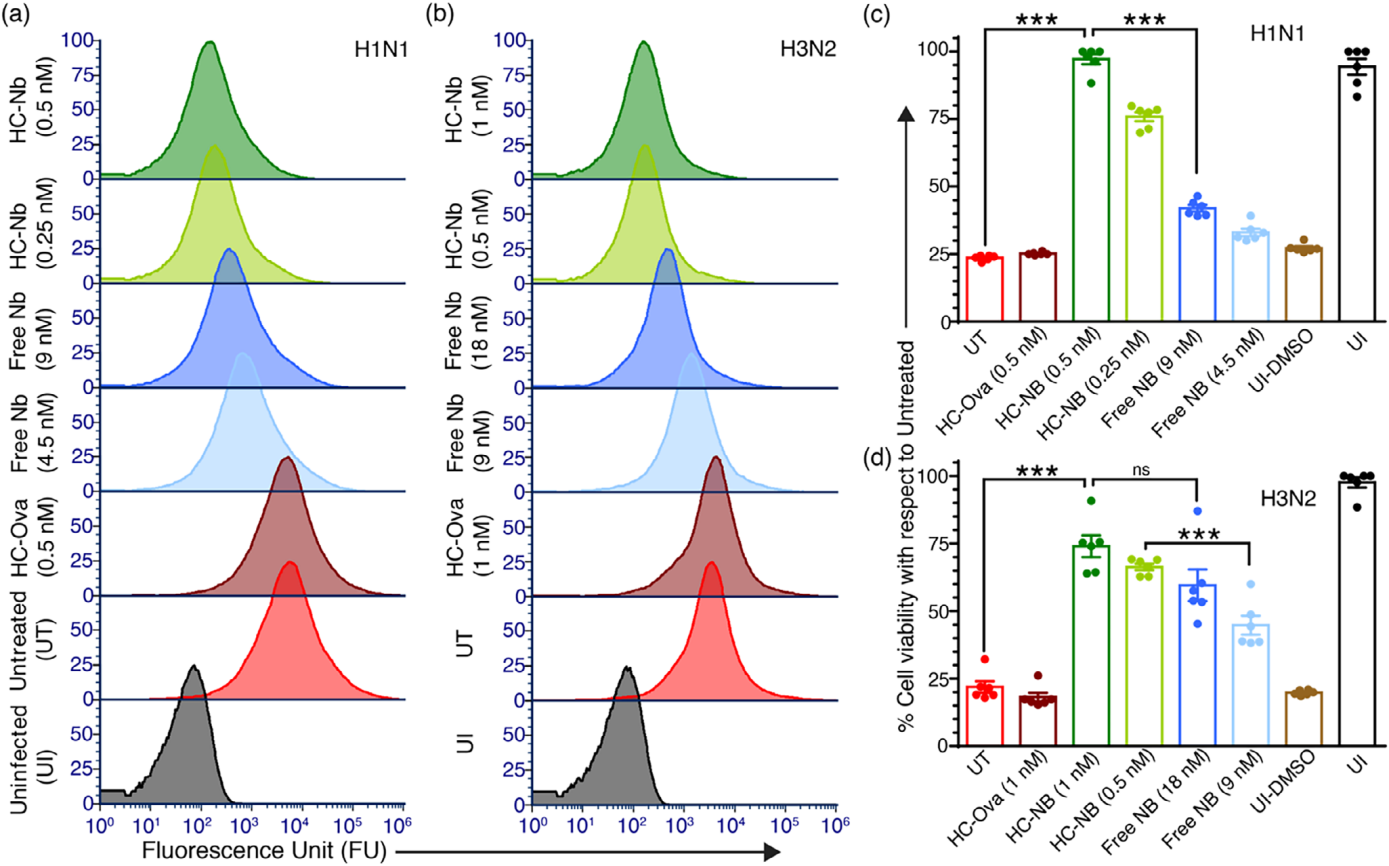
HC–Nb construct maintains potent antiviral activity and limits infection by porcine‐adapted influenza A viruses (IAV). a) 1‐h postinfection, flow cytometry reveals that the HC–Nb construct more effectively blocks H1N1 virion entry into porcine epithelial cells than free nanobodies. Controls include the HC scaffold presenting a non‐target peptide (ovalbumin), untreated virions, and uninfected cells. b) A comparable but slightly greater inhibitory effect is observed for H3N2 subtype, demonstrating the robust cross‐subtype efficacy. c,d) MTT viability assays at 24 h postinfection confirm that HC–Nb treatment significantly improves cell survival for H1N1 c) and H3N2 d) relative to free nanobodies, suggesting its enhanced antiviral protection (DMSO‐treated uninfected cells serve as a positive control for cell death; bars show mean and standard error; ns indicates observed effect is statistically non‐significant, ^***^ *p* < 0.0001).

Following the experimental protocol established for murine models, we assessed the antiviral efficacy and cytoprotective effects of the HC–Nb construct against porcine‐adapted IAV subtypes. Specifically, porcine‐adapted H1N1 virions were pre‐incubated with either 0.5 nm HC–Nb or an equivalent 9 nm concentration of free nanobody prior to infection of porcine epithelial cells. Flow cytometry analysis shows that treatment with 0.5 nm HC–Nb could reduce the mean fluorescence intensity to ≈80 RFU, closely matching the uninfected control (≈10 RFU) and corresponding to >97% inhibition of viral entry (**Figure** **6**a; Figure S14a, Supporting Information). In contrast, pretreatment with free nanobody yields a higher fluorescence signal (≈230 RFU), equating to ≈92.3% inhibition. A similar trend was observed for the H3N2 subtype, where the treatment with 1 nm HC–Nb achieves 96% inhibition, while 18 nm free nanobody achieves only ≈87.5% inhibition (Figure 6b; Figure S14b, Supporting Information). These results are consistent with the performance trends observed in the murine models, suggesting the robust and cross‐species efficacy of the HC–Nb construct.

To further evaluate cytoprotective effects under physiologically relevant conditions, we performed MTT assays using a similar experimental methodology. Cells infected with untreated virus or virus pre‐treated with the non‐binding HC–Ova control exhibit ≈25% loss in viability for both IAV subtypes, reflecting significant virus‐induced cytopathic effects. In contrast, pre‐treatment with 0.5 nm HC–Nb preserved cell viability at ≈97.5%, comparable to uninfected controls, whereas treatment with an equivalent concentration of free nanobody (9 nm) restored viability to only ≈42.5% in the H1N1 infection model (Figure 6c). This indicates greater than 55% improvement in cell survival conferred by the HC–Nb construct, likely attributable to its multivalent architecture, which enables simultaneous engagement of multiple HA trimers and more effective blockade of virus–host interactions. We observed consistent findings for the H3N2 infection model, wherein the treatment with HC–Nb at 1 nm maintains ≈75% cell viability, while free nanobody at 18 nm yields only ≈45% viability, reflecting a 30% improvement in cytoprotection (Figure 6d).

To further examine the nature of the cell death, we assessed oxidative stress, a well‐established cellular response preceding cell death. MDCK cells were infected with porcine H1N1 virus pretreated under different conditions (untreated, HC‐Ova, HC‐Nb, HC‐UHA2, free nanobody, or free UHA2 aptamer). Two hours postinfection, untreated and HC–Ova–treated groups exhibited significantly increased ROS production, reaching around twofold above baseline and comparable to the DMSO control (Figure S15, Supporting Information). By contrast, HC‐Nb and HC‐UHA2 treatment markedly suppressed ROS production, restoring levels close to baseline. Free nanobody and aptamer counterparts produced only minimal reductions in ROS, consistent with their weaker neutralization capacity. These findings provide additional evidence that HC‐DDN mediated multivalent display enhances antiviral efficacy by both reducing infection and thus mitigating virus‐induced cellular stress.

To further benchmark HC–Nb‐based platform, we compared its efficacy with nanobody‐functionalized gold nanoparticles (AuNP–Nb), synthesized as described in the Materials and Methods. Against porcine H1N1, treatment with 0.5 nm HC–Nb or 0.5 nm AuNP–Nb resulted in 96.5% and 86.4% inhibition, respectively, while free nanobody and free AuNP achieved only 70.5% and 59.1% inhibition (Figure S16a,c, Supporting Information). For porcine H3N2, treatment with 1 nm HC–Nb and 1 nm AuNP–Nb achieved 95.5% and 86.5% inhibition, respectively, compared to 70% and 60% with free nanobody and free AuNP (Figure S16b,d, Supporting Information). Notably, AuNPs alone exhibited modest antiviral activity, consistent with prior observations that inorganic nanoparticle cores can contribute to nonspecific antiviral effects.^[^ ^57^, ^58^, ^59^ ^]^

To access if AuNPs can be used as an alternative to HC‐DDN, we compared their safety profiles by assessing cytotoxicity using an MTT assay at 24 and 48 h post‐treatment. AuNP exposure reduced host cell viability by ≈25%, however HC–DDN scaffolds showed no detectable effect on viability (Figure S17, Supporting Information), suggesting that HC‐DDN is a safer carrier as compared to AuNP. Taken together, these results demonstrate that HC–DDN scaffolds not only provide superior antiviral potency compared to AuNPs but also circumvent the cytotoxicity associated with inorganic nanoparticles, highlighting their translational potential as a safe and biocompatible antiviral platform.

Collectively, these findings highlight the functional advantage of the HC–Nb's multivalent design, which enhances effective epitope engagement and neutralization across diverse IAV subtypes. The superior antiviral and cytoprotective performance observed in both murine and porcine models places the HC–Nb construct as a promising candidate for further development as a broad‐spectrum, next‐generation antiviral therapeutic.

## 3 Conclusion and Future Work

Herein, we developed and systematically validated an effective multivalent antiviral platform based on a honeycomb‐shaped designer DNA nanostructure functionalized with either high‐affinity nanobodies or broad‐spectrum DNA aptamers. By precisely arranging multiple binding ligands to mimic the native spatial distribution of IAV HA trimers, this platform leverages cooperative multivalent interactions to dramatically enhance binding avidity and thus neutralization potency compared to free forms of the monovalent ligands. Surface plasmon resonance analyses confirm that both nanobody‐ and aptamer‐functionalized HC‐DDN constructs have achieved sub‐picomolar to picomolar binding affinities, in stark contrast to the nanomolar affinities observed for their free counterparts, when tested against different IAV subtypes.

In vitro assays using murine and porcine epithelial cell models demonstrate that these multivalent constructs not only suppress viral entry more effectively but also provide superior cytoprotection at substantially lower nanomolar concentrations. For example, compared to free nanobodies, the HC–Nb construct reduces virus‐induced cytopathic effects by 35–45% in murine models across both H1N1 and H3N2 subtypes. Notably, the HC–Nb construct consistently outperforms the HC–Apt construct in both infection inhibition and cell viability preservation, suggesting the intrinsic advantage of nanobodies as robust, high‐affinity binders for viral neutralization. Extending these findings to a clinically relevant porcine model further demonstrated a 30–55% improvement in cytoprotective effects across H1N1 and H3N2 subtypes, validating the cross‐species efficacy of the HC–Nb construct and suggesting its potential as a broadly applicable therapeutic strategy for combating rapidly evolving influenza subtypes.

While detailed studies on administration routes, immunogenicity, scalability, pharmacokinetics, tissue biodistribution, in vivo therapeutic efficacy remain beyond the scope of the present work, existing evidence of DNA nanostructure biocompatibility,^[^ ^60^ ^]^ the low immunogenicity of nanobodies, and demonstrated scalability of DNA nanostructure assembly support the feasibility of further development. Future efforts will focus on formulation optimization, pharmacokinetic evaluation, and preclinical validation, with the long‐term goal of translating DDN‐based antivirals into clinically relevant therapies. Importantly, the modular and programmable nature of the HC‐DDN scaffold enables straightforward extension to other class I fusion viruses, including members of the paramyxovirus family. More broadly, this work establishes a foundation for the rational design of next‐generation antiviral agents using programmable DNA nanostructures. The comparative framework introduced here offers a practical blueprint for evaluating and optimizing both nucleic acid– and protein–based therapeutics within a unified spatial context, enabling customized strategies against diverse viral pathogens and rapidly emerging clinical threats.

## Conflict of Interest

The authors declare no conflict of interest.

## Author Contributions

S.U., A.D., and D.G. contributed equally to this work. S.U. and X.W. conceived the study, including the design of the nanobody, aptamer, and HC‐DDN selection. S.U., A.D., and D.G. designed and performed experiments, carried out formal analyses, and conducted investigations. C.C., V.M., L.Z., J.D., and Y.F. provided critical resources. S.U. curated data, prepared visualizations, and wrote the original draft. X.W. supervised the study and acquired funding. All authors discussed the results, revised the manuscript, and approved the final version.

## Supporting information



Supporting Information

Supplemental Movie 1

## Data Availability

The data that support the findings of this study are available in the supplementary material of this article.
