# Supplementary material for: DNA Nanostructure‐Templated Multivalency Enables Broad‐Spectrum Virus Inhibition
Source: Adv Sci (Weinh). 2025 Nov 21;13(14):e13710. doi: 10.1002/advs.202513710 (PMC12970221; doi:10.1002/advs.202513710)
Supplement: Supplementary file 1 — Supporting Information [file ADVS-13-e13710-s002.pdf]

Supporting Information

DNA Nanostructure-Templated Multivalency Enables Broad-Spectrum Virus Inhibition

Saurabh Umrao[1,2,3,†,*], Abhisek Dwivedy[1,2,3,†], Dhanush Gandavadi[1,2,3,†], Chi Chen[4], Lifeng Zhou[5], Jinwei Duan[6], Vineetha Mareddy[3,4], Ying Fang[3,4], Xing Wang[1,2,3,7,8,*]

[1] Department of Bioengineering, University of Illinois at Urbana-Champaign, Urbana, IL, USA

[2] Nick Holonyak Jr. Micro and Nanotechnology Laboratory, University of Illinois at Urbana-Champaign, Urbana, IL, USA

[3] Carl R. Woese Institute for Genomic Biology, University of Illinois at Urbana-Champaign, Urbana, IL, USA

[4] Department of Pathobiology, University of Illinois at Urbana-Champaign, Urbana, IL USA

[5] Department of Advanced Manufacturing and Robotics, Peking University, Beijing, China

[6] Department of Chemistry and Materials Science, School of Sciences, Chang'an University, Xi'an, Shaanxi, China

[7] Department of Chemistry, University of Illinois at Urbana-Champaign, Urbana, IL, USA

[8] Cancer Center at Illinois, University of Illinois at Urbana-Champaign, Urbana, IL, USA

† Contributed equally to this work

* Saurabh Umrao (usaurabh@illinois.edu), Xing Wang (xingw@illinois.edu)

Materials and Methods.

Coarse-grained oxDNA simulation setup.

We prepared the initial oxDNA design files using the oxView interface and optimized the structures through the NANOBASE.org server to perform energy relaxation. The relaxation was conducted first with the CPU-based algorithm using 10^9 MD steps, then refined using the CUDA (GPU) backend to improve computational efficiency. Following relaxation, we generated the required topology and configuration files and submitted them to the oxDNA.org web server to run coarse-grained molecular dynamics simulations. All simulations were performed on a GPU-enabled high-performance computing server at a temperature of 20 °C and a sodium ion concentration of 0.15 M. The system was simulated for 10^6 MD steps using an integration timestep of 0.001 simulation units. After simulation completion, we used the integrated oxView browser-based visualizer to inspect structural trajectories and analyze dynamic behavior in detail.

Modelling, docking and minimization of nanobody HA complex.

The amino acid sequence of the nanobody construct[1], containing of two variable heavy-chain domains connected by a flexible $(GGGGS)_3$ linker, was submitted to the Robetta protein structure prediction server[2]. Robetta was used in de novo structure prediction mode to generate three-dimensional atomic models of the construct. The top-ranked predicted structural model was downloaded in PDB format and used for downstream structural analyses and visualization. The HA trimer structure **(PDB ID: 6FYT)** was downloaded from the Protein Data Bank, and the monomer structure was extracted and cleaned using PyMol. The modeled nanobody structure was then docked onto the extracted HA monomer using HADDOCK 2.4 with positional restraints of 1.5Å. Based on previously reported interactions of SD36 and SD38 nanobodies with HA protein[1], the best docking model was selected. One-, two-, and three-nanobody complexes with the HA trimer were subsequently generated by sequential superposition in PyMOL. For comparison, the HA trimer structure bound to sialic acid **(PDB ID: 7VDF)** was also retrieved. All structures were energy-minimized using the YASARA minimization server and further analyzed with the YASARA scene viewer[3]. Final structural visualization and analysis were performed using PyMOL and UCSF ChimeraX[4].

Experimental Section.

Bivalent multidomain nanobody design and synthesis.

We repurposed a bivalent multidomain nanobody construct (SD38–SD36)[1] composed of the following amino acid sequence: GHHHHHHEVQLVESGGGLVQPGGSLRLSCAVSISIFDIYAMDWYRQAPGKQRDLVATSFRDGS TNYADSVKGRFTISRDNAKNTLYLQMNSLKPEDTAVYLCHVSLYRDPLGVAGGMGVYWGKGAL VTVSSAAA(GGGGS)$_3$EVQLVESGGGLVQAGGSLKLSCAASGRTYAMGWFRQAPGKEREFVAH INALGTRTYYSDSVKGRFTISRDNAKNTEYLEMNNLKPEDTAVYYCTAQGQWRAAPVAVAAEYE FWGQGTQVTVS. This construct includes an N-terminal GHHHHHH tag for DNA conjugation and a flexible AAA(GGGGS)$_3$ linker connecting the two nanobody domains. The bivalent nanobody gene was custom synthesized and expressed by Bon Opus Biosciences, New Jersey.

Materials.

All DNA oligonucleotides for assembling the HC-DDN were purchased from Integrated DNA Technologies (IDT) and used without further purification. This included the DNA sequence for nanobody conjugation (GCTGCTATTGCGTTT-DBCO), the UHA2 aptamer[5] (GCTGCTATTGCGTCCGTTTTAGATTGGCCTTGCTATCACCCAAAACCGTTTAAAGCTGACCA CGTGACGCTTCATCCGT), and the FAM-labeled V46 aptamer[6] (5′-FAM-GCTGCTATTGCGTCCGTTTTACTGCACACGACACCGACTGTCACCATCACCTCGGCGCA). The DNA sequences used to prepare HC-DDN nanostructure is listed in Table S1. The OVA control peptide, comprising a DBCO-azidohexanoic acid linker attached to the SIINFEKL epitope (GCTGCTATTGCGTTT-DBCO-azidohexanoic acid-SIINFEKL), was custom synthesized and HPLC-purified by Biomers Inc. Murine-adapted influenza A virus strains H1N1 (VR-95™, strain A/PR/8/34; PR8) and H3N2 (VR-1881, strain A/Wisconsin/67/2005) were obtained from ATCC, each supplied as viral stocks with a titer of 2.1 × 10^8 $CEID_{50}$/mL.

HC-DDN structure synthesis and PEG-Purification Process.

We assembled the honeycomb-shaped DNA nanostructure (HC-DDN) by mixing 20 nM M13 scaffold strand (p7249) with 200 nM single-stranded DNA staple strands in 1× TAE-Mg^{2+} buffer (pH 7.5), containing 40 mM Tris-acetate, 2 mM EDTA, and 16 mM magnesium chloride, in a 0.2 mL PCR tube (USA Scientific). The assembly mixture was annealed in a thermal cycler (Bio-Rad T100 PCR Thermal Cycler) using a programmed temperature ramp: initial heating at 80 °C for 5 min; gradual cooling from 80 °C to 60 °C at a rate of 1 °C every 5 min; further cooling from 60 °C to 34 °C at a rate of 5 °C every 45 min; and finally, cooling from 34 °C to 4 °C at a rate of 1 °C every 5 min. The complete annealing process lasted approximately 44 hours. After annealing, we purified the assembled HC-DDN (20 nM) by adding an equal volume of a PEG solution containing

15% (w/v) PEG 8000, 5 mM Tris, 1 mM EDTA, and 505 mM NaCl. After thorough mixing, the mixture was chilled on ice for 5 minutes and then centrifuged at 16,000 × g at 4 °C for 25 minutes. The supernatant was carefully removed, and the pellet was gently resuspended in 1× TAE/Mg^{2+} buffer. To allow complete re-dissolution of the nanostructure, the sample was incubated at room temperature overnight. This precipitation and resuspension cycle was repeated twice more to ensure effective removal of excess staple strands. The final purified HC-DDN was stored at 4 °C until further experiments.

Agarose gel electrophoresis, and atomic force microscopy (AFM) imaging of the HC-DDN construct.

We analyzed the HC-DDN samples by running 1% agarose gel electrophoresis (AGE) prepared with 1× TAE-Mg^{2+} buffer. To enable direct visualization, the gel was pre-stained with SYBR Green dye (Thermo Fisher Scientific). Electrophoresis was carried out at a constant field strength of 40 V/cm for 2 hours. Following run, the gel was imaged using a GelDoc system (Thermo Fisher Scientific) to assess the structure formation.

To prepare samples for AFM, we deposited a 5 µL drop of the ~20 nM unpurified HC-DDN solution onto a freshly cleaved mica substrate and allowed it to adsorb for 5 minutes. The mica surface was then gently rinsed with 1 mL of deionized water and dried using compressed air. We performed AFM imaging in air under tapping mode using a Cypher AFM system (Asylum Research) equipped with a 4XC AFM probe (OPUS, MikroMasch).

Serum and pH Stability of HC-DDN nanostructures.

To assess serum stability, 1 nM HC–DDN constructs were incubated in 10% mouse serum at 37 °C for varying time points (0, 6, 12, and 24 h). Samples were analyzed by 1% agarose gel electrophoresis (AGE), as described above, to qualitatively and quantitatively evaluate the degradation profile.

To assess pH stability, 1 nM HC–DDN constructs were incubated in 1× TAE buffer adjusted to pH values ranging from 3.0 to 9.0. Samples were analyzed by 1% AGE to qualitatively evaluate structural stability under each condition.

Synthesis and Purification of DNA-nanobody Conjugates.

For DNA–nanobody conjugation, 4-methoxyphenyl 2-azidoacetate was first dissolved in 99% acetonitrile to prepare a 15 mM stock solution. To modify the nanobody with azide groups, a 24 µL

aliquot of this stock solution (corresponding to a 120-fold molar excess, yielding a final azide concentration of approximately 4200 μM) was added to 80 μL of a 35 μM glycine–His-tagged nanobody solution prepared in 200 mM HEPES buffer (pH 7.4). The exact nanobody concentration was verified at this stage by measuring absorbance at 280 nm using a Nanodrop spectrophotometer. The reaction mixture was incubated at 4 °C for 1 to 3 days in the dark, with incubation time optimized for each protein. After incubation, the azide-modified nanobody was purified by centrifugation through a 3 kDa Amicon centrifugal filter unit and washed three times with 200 μL of 1× PBS to remove unreacted azide reagent, then adjusted to a final volume of 80 μL in 1× PBS. The purified azide-modified nanobody was then mixed with an equal volume of a 35 μM DBCO-modified DNA solution in 1× PBS to initiate the conjugation reaction, which proceeded at 4 °C for 24 hours. The resulting protein–DNA conjugate was purified using a 30 kDa Amicon centrifugal filter to remove unreacted DNA strands and washed three additional times with 1× PBS to ensure thorough removal of excess DNA. The purified nanobody–DNA conjugate was collected and quantified using the Nanodrop (ssDNA mode) and the final yield was calculated according to the formula: yield = (new concentration × new volume) / (original concentration × original volume).

Thiolated DNA design and pretreatment.

A thiol-modified probe DNA (sequence: 5′-CGGACGCAATAGCAGCTTT-3′, with a 3′-ThioMC3 modification) was designed to be complementary to the single-stranded DNA conjugated to the nanobody. The thiolated DNA was synthesized by Integrated DNA Technologies (IDT) and reduced using 200 molar equivalents of tris(2-carboxyethyl)phosphine hydrochloride (TCEP). The reduced DNA was then purified using a NAP-25 column (Cytiva) pre-equilibrated with nuclease-free water. The final DNA solution (1.5 mL in nuclease-free water) was collected, and its concentration was determined by a NanoDrop One spectrophotometer (Thermo Scientific).

DNA modification on gold nanoparticles.

DNA functionalization of gold nanoparticles (AuNPs) was performed following a previously published protocol[7]. Briefly, thiolated oligonucleotides (1000-fold molar excess) were mixed with an aqueous solution of 20 nm AuNPs (particle concentration ~7×10^{11} particles/mL; Ted Pella) and incubated for 12 h at room temperature. The mixture was then transferred into 10 mM phosphate buffer (NaH_2PO_4/Na_2HPO_4, pH 7.4). Salt aging was carried out by gradually increasing the NaCl concentration to 0.3 M in three steps, each step at 8 h intervals. To remove unbound DNA, the AuNPs were centrifuged at 13,000 rpm for 30 min, and the supernatant was discarded.

The washing step was repeated four times, and the resulting DNA-functionalized AuNPs were stored at 4 °C until use.

Surface Plasmon Resonance (SPR) Analysis.

All surface plasmon resonance (SPR) measurements were performed using a Biacore T200 system (GE Healthcare, Uppsala, Sweden) operated with BIAcore T200 control software. Whole IAV (H1N1 and H3N2) and SARS-CoV-2 virions were immobilized on individual flow cells (FC2, FC3, and FC4, respectively) of a research-grade CM5 S-Series sensor chip via standard amine coupling chemistry. Briefly, the carboxymethylated dextran matrix on the chip surface was activated with a 420-second injection of 1:1 mixture of 50 mM N-ethyl-N′-(3-dimethylaminopropyl) carbodiimide (EDC) and 50 mM N-hydroxysuccinimide (NHS) at a flow rate of 5 μL/min. Virions were diluted to ~1 × 10^6 particles/mL in 10 mM sodium acetate buffer (pH 5.0) and injected to immobilize H1N1 on FC2, H3N2 on FC3, and SARS-CoV-2 on FC4. Flow cell FC1 was reserved as a reference and underwent the same activation and deactivation steps without virion immobilization to account for bulk refractive index shifts and non-specific binding. Residual active esters were quenched by a 600-second injection of 1 M ethanolamine (pH 8.5) at 5 μL/min across all flow cells. SPR binding measurements were performed at 25 °C using filtered PBS with 0.05% Tween-20 as the running buffer. Free nanobodies, free aptamers, and multivalent constructs (HC–Nb, HC–Apt, and HC–V46) were injected at concentrations spanning the picomolar to micromolar range, with an association phase of 120 s and a dissociation phase of 600 s at a flow rate of 30 μL/min. Following each injection, the sensor surface was regenerated with a 60-second pulse of 25 mM NaOH at 100 μL/min, followed by a 5-minute stabilization interval before the next cycle. Binding response data (sensorgrams) were recorded in real time. Kinetic parameters—including the association rate constant (k_a), dissociation rate constant (k_d), and the equilibrium dissociation constant (K_D, where $K_D = k_d/k_a$)—were determined by globally fitting the association and dissociation phases using BIAevaluation Software 4.0.1 (GE Healthcare).

Swine virus propagation and quantification.

We cultured Madin–Darby Canine Kidney (MDCK) cells in 24-well plates by seeding 2 × 10^5 cells per well in 1 mL of Minimum Essential Medium (MEM; Gibco, Carlsbad, CA) supplemented with 10% fetal bovine serum (FBS; Sigma, St. Louis, MO), sodium bicarbonate ($NaHCO_3$; Invitrogen, Carlsbad, CA), MEM vitamins (Invitrogen), L-glutamine (Invitrogen), and a standard antibiotic mix (penicillin, streptomycin, and gentamicin; Gibco). The cells were incubated overnight at 37 °C in a 5% CO_2 atmosphere to allow them to reach approximately 80–95% confluency.

For infection, we removed the culture medium and washed the cell monolayers twice with sterile phosphate-buffered saline (PBS) to eliminate residual serum. We then inoculated the MDCK cells with influenza A virus strains A/sw/NC/18161/02 (H1N1) or A/sw/TX/4199-2/98 (H3N2) at an MOI of 0.02, using 200 µL per well of infection medium composed of MEM supplemented with 0.3% bovine serum albumin (BSA; Sigma), $NaHCO_3$, MEM vitamins, L-glutamine, and antibiotics. The cells were incubated at 37 °C for 1 hour, with gentle rocking every 15 minutes to ensure even viral adsorption. After this period, we removed the inoculum and added fresh infection medium containing 1 µg/mL TPCK-treated trypsin (Worthington, Columbus, OH) to promote viral replication. The infected cultures were then incubated at 37 °C for 2 to 3 days and monitored daily for cytopathic effects (CPE). When substantial CPE was observed, typically at 48 hours post-infection, we collected the culture supernatants, purified by centrifugation at 3,000 × g for 10 minutes at 4 °C to remove debris, aliquoted the clarified virus stocks, and stored at −80 °C.

To determine the virus titer, we performed serial ten-fold dilutions of the harvested virus and used these to infect MDCK cells grown in 96-well plates. After 1 hour of incubation to allow viral adsorption, we replaced the inoculum with fresh infection medium containing 1 µg/mL TPCK-trypsin and continued incubation for 48 hours. The infected cells were then fixed using an equal mixture of methanol and acetone pre-chilled to −20 °C, and subsequently incubated at −20 °C for 20–60 minutes. After incubation, the fixing solution was removed, and the plate was air-dried. For immunostaining, we incubated the fixed cells with an anti-influenza A virus nucleoprotein monoclonal antibody as the primary antibody at 37 °C for 1 hour. After washing three times with 1x PBS, we added Alexa Fluor 488–conjugated goat anti-mouse IgG (Jackson ImmunoResearch, West Grove, PA) diluted 1:800 in 1x PBS and incubated for another hour at room temperature. Finally, cells were washed thoroughly with 1x PBS and examined under a fluorescence microscope to visualize and quantify infection levels for viral titration.

Flow cytometry analysis for assessing viral infection.

We utilized a previously reported assay to determine viral infection in host cells using flow cytometry[8]. More specifically, we prepared murine- and swine-adapted H1N1 and H3N2 influenza A virus stocks (1×10^7 particles per condition) and pre-treated them with either free nanobodies or nanobodies displayed on the HC-DDN. As specificity controls, we included virus samples incubated with HC-DDN presenting an OVA peptide and samples with no treatment. After allowing the nanobody binding to proceed for 1 hour and 45 minutes, we supplemented each preparation with HC-DDN bearing FAM-labeled V46 aptamers and continued incubation for an additional 15 minutes to permit aptamer attachment.

Following pretreatment, we exposed Madin–Darby Canine Kidney (MDCK) cells to the prepared virus suspensions. For each sample, we used 10^4 MDCK cells (MOI of 1:1000) resuspended in flow cytometry buffer composed of 20 mM HEPES (pH 7.4), 150 mM NaCl, 12.5 mM $MgCl_2$, and 10% fetal bovine serum, adjusting the total reaction volume to 100 µL. Virus-free cells treated identically served as negative controls for baseline correction of background fluorescence. The infection step proceeded for 60 minutes, after which cells were washed twice with fresh buffer to remove excess virus and free reagents.

Fluorescence measurements were performed on an Attune NxT Flow Cytometer (Thermo Fisher, model A24858), equipped with both 488 nm (blue) and 637 nm (red) excitation lasers. FAM fluorescence signals were collected using the BL1 channel with a 530/30 nm bandpass filter. Since only one fluorophore (FAM) was used in this experiment, compensation was not applied. For data integrity, we gated collected events by forward and side scatter to isolate viable cells, then applied a doublet discrimination gate (forward scatter area versus height) to acquire only single-cell events. FAM fluorescence intensity was quantified specifically for the single-cell population. For comparative analysis, we reported the median fluorescence intensity (MFI) of the FAM signal as an indicator of relative viral binding and/or internalization in each treatment group.

Cell viability assessment assay.

We evaluated cell viability using an MTT colorimetric assay conducted 24 and 48 hours after viral infection or nanoparticle (HC-DDN or AuNP) treatments. To detect any cytotoxic effects of nanoparticles, cells were treated with HC-DDN or AuNP at appropriate concentrations and allowed to proliferate for 24 or 48 hrs. To detect cytotoxic effect of infection, virus treatment and cell infection procedures were identical to those detailed in the Flow Cytometry Analysis section, with each infection carried out in a total volume of 100 µL. One hour following infection, cells were washed twice with fresh flow cytometry buffer to remove residual virus and then overlaid with standard culture medium.

At the 24-hour and 48-hour time point, we added 10 µL of freshly prepared 12 nM MTT solution (dissolved in 1× PBS, pH 7.2) directly to each well and incubated the plates for 3 hours at 37 °C to allow viable cells to convert MTT into formazan crystals. To solubilize the resulting formazan, we then added 100 µL of a lysis solution containing 0.01 M HCl, 12% (w/v) SDS, and 20% (v/v) DMSO, and incubated the plates for an additional 3 hours at room temperature with gentle mixing to ensure complete dissolution. After solubilization, we measured the absorbance of each well at 570 nm using a microplate spectrophotometer, quantifying the intensity of the dissolved formazan

as a readout for metabolic activity. Absorbance values obtained from uninfected control wells were defined as representing 100% cell viability. Viability percentages for infected cell samples were then calculated relative to this control baseline.

Reactive oxygen species (ROS) detection assay.

MDCK cells were infected with H1N1 virus under different treatment conditions (untreated, HC-Ova, HC-Nb, HC-UHA2, free Nanobody, and free UHA2 aptamer, as described earlier). Cells treated with DMSO served as a positive control for ROS induction, as reported in previous studies[6,7]. ROS detection was performed 2 h post-infection using a commercial ROS detection kit (ab186029, Abcam, USA), following the manufacturer's protocol. Fluorescence was quantified by a microplate spectrophotometer, and relative ROS levels were calculated using uninfected samples as the baseline control.

Statistical Analyses.

All statistical evaluations were carried out using GraphPad Prism version 10.4.1. Differences between experimental groups in flow cytometry and MTT assays were assessed using a two-tailed unpaired Student's t-test with Welch's correction to account for unequal variances. A p-value below 0.0001 was deemed statistically significant. Each experiment was independently repeated at least three times, using separate cell culture preparations to account for replication. The number of replicates per experiment is mentioned as "n" in the figure legends.

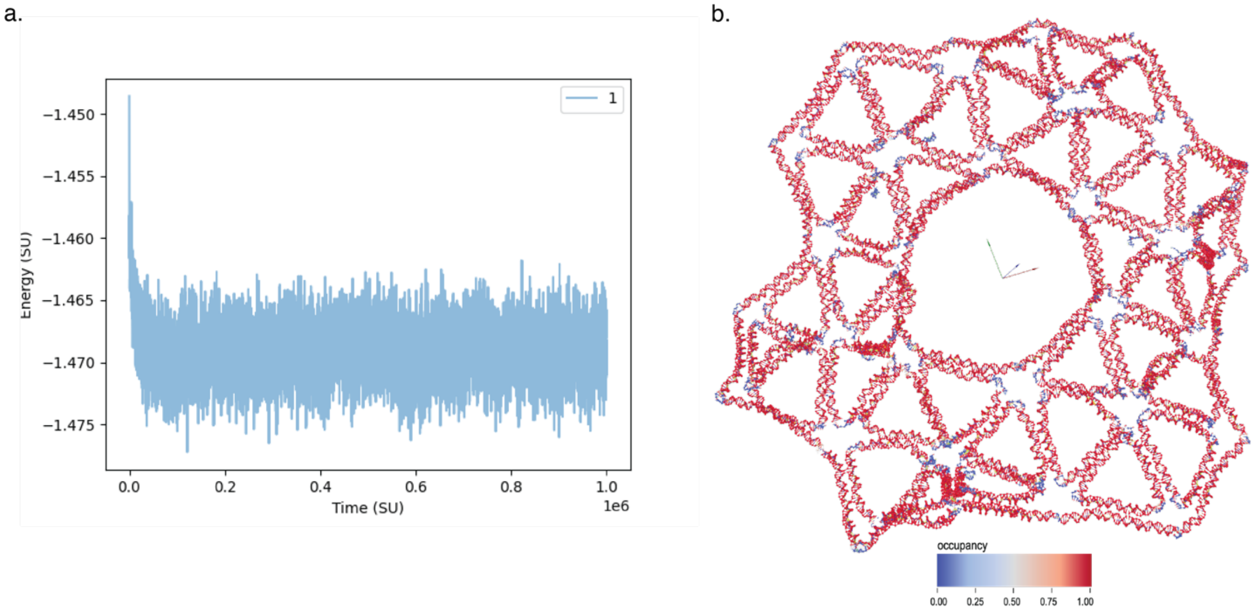

Figure S1. Potential energy and bond occupancy of the HC-DDN nanostructure during simulation. (a) Potential energy profile of the nanostructure showing its progression from an unequilibrated initial state to a fully equilibrated state. The system stabilizes as the energy converges and fluctuates around a mean value of –1.470 simulation energy units per particle. (b) Bond occupancy map of the nanostructure, where dark red regions represent double-stranded DNA, and blue regions indicate single-stranded segments, including those within the trimeric units and T-bulges.

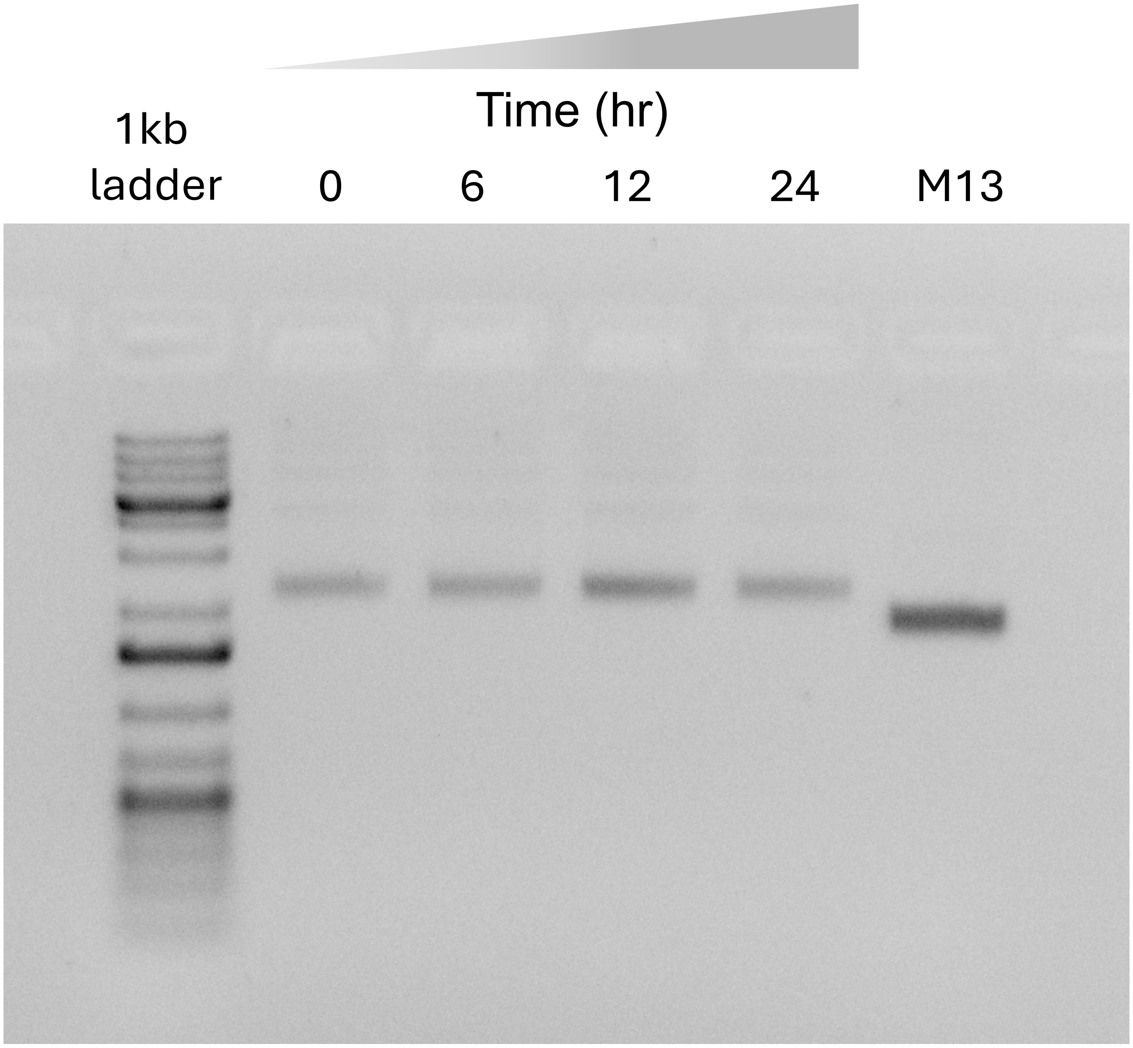

Figure S2. Stability and degradation profile of HC-DDN in mouse serum. HC-DDN nanostructures were incubated in 10% mouse serum at 37 °C and sampled at multiple time points up to 24 h. Samples analyzed by 1% agarose gel electrophoresis (AGE) show that HC-DDN retained its structural integrity under physiological conditions, with minimal to no detectable degradation during the 24 h incubation.

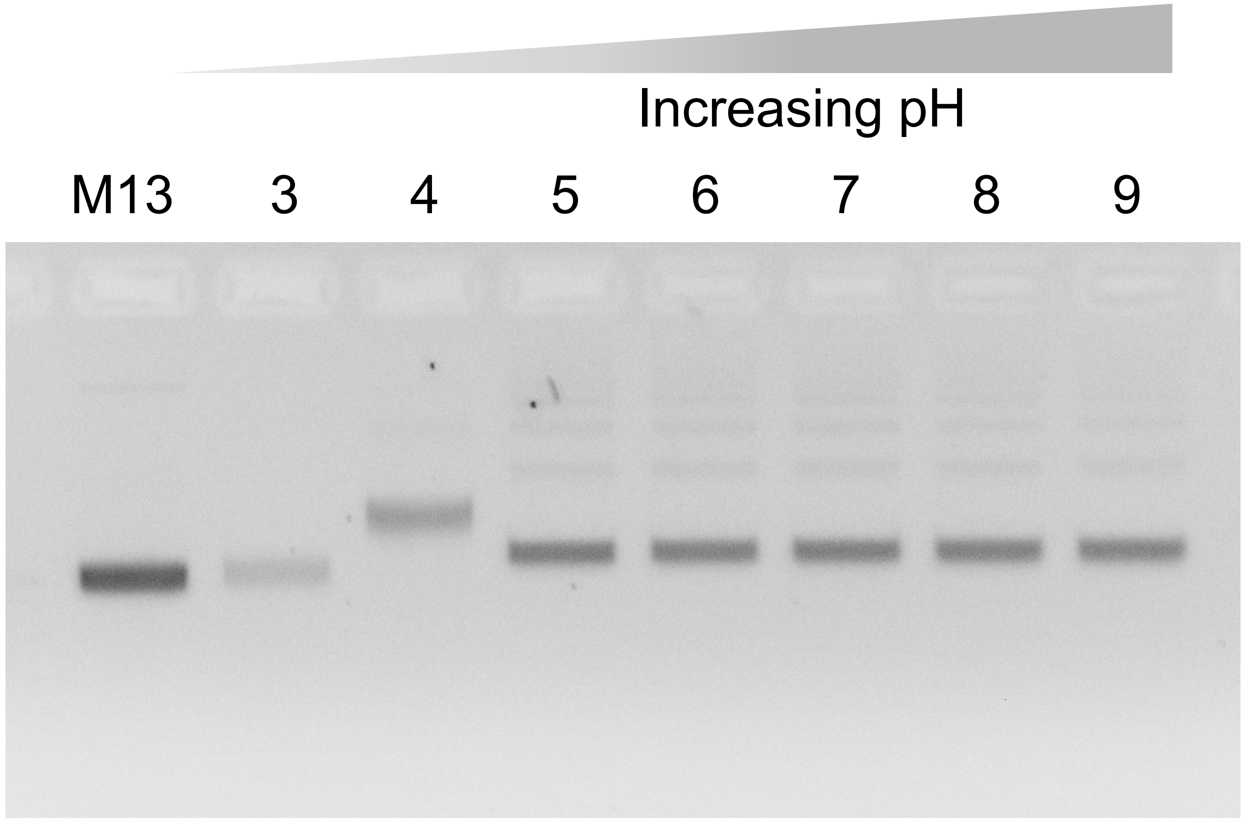

Figure S3. pH stability of HC-DDN nanostructures. HC-DDN was incubated in 1x TAE buffers spanning pH 3.0–9.0 and analyzed by 1% agarose gel electrophoresis (AGE). The M13 DNA scaffold strand was included in the first lane as a control. HC-DDN remained intact from pH 5.0 to 9.0, exhibited a modest mobility shift at pH 4.0 (consistent with partial protonation effects), and showed moderate degradation at pH 3.0.

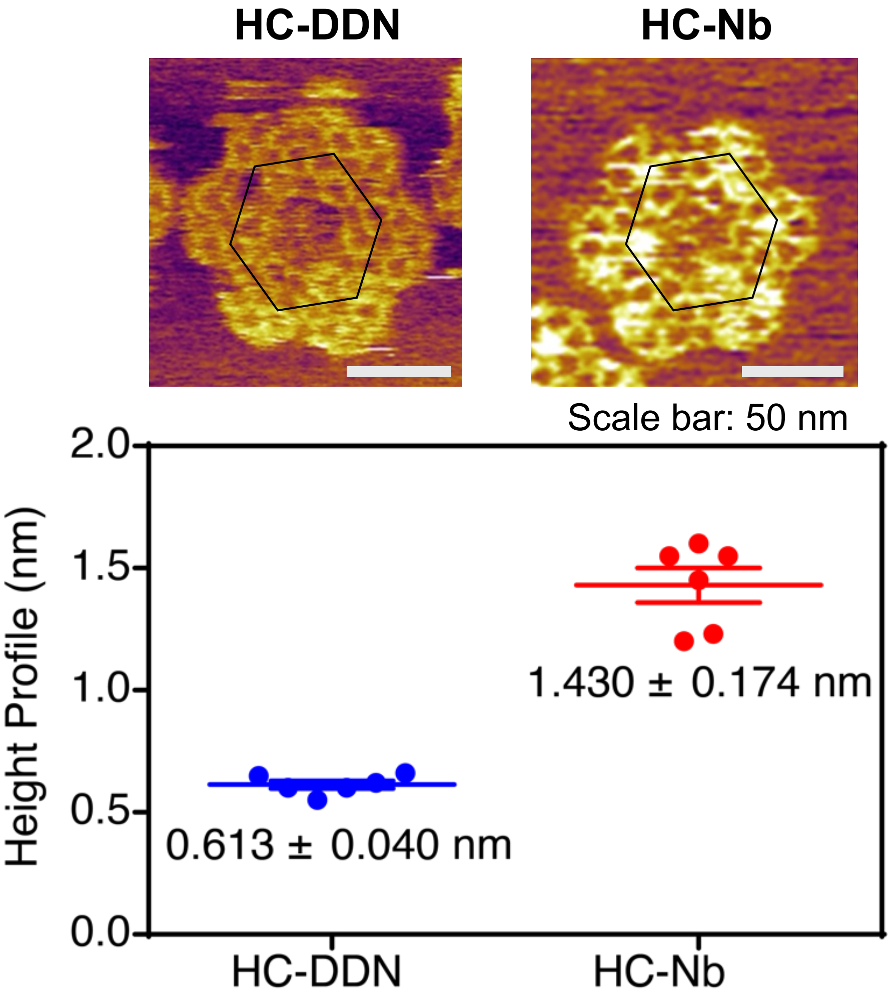

Figure S4. Atomic force microscopy (AFM) characterization of nanobody conjugation to HC-DDN. AFM was used to compare control HC-DDN scaffolds with nanobody-conjugated HC–Nb constructs under matched imaging conditions. The top panel shows representative AFM micrographs of HC-DDN and HC–Nb acquired at identical magnification, confirming that overall structural integrity is maintained following nanobody conjugation. The bottom panel shows representative height profiles extracted along honeycomb vertex edges, which reveal increased vertex height in HC–Nb relative to control HC-DDN. Quantitative analysis of vertex height distributions indicates a significant increase for HC–Nb (1.430 ± 0.174 nm) compared to HC-DDN (0.613 ± 0.040 nm), consistent with successful nanobody attachment at designated display sites.

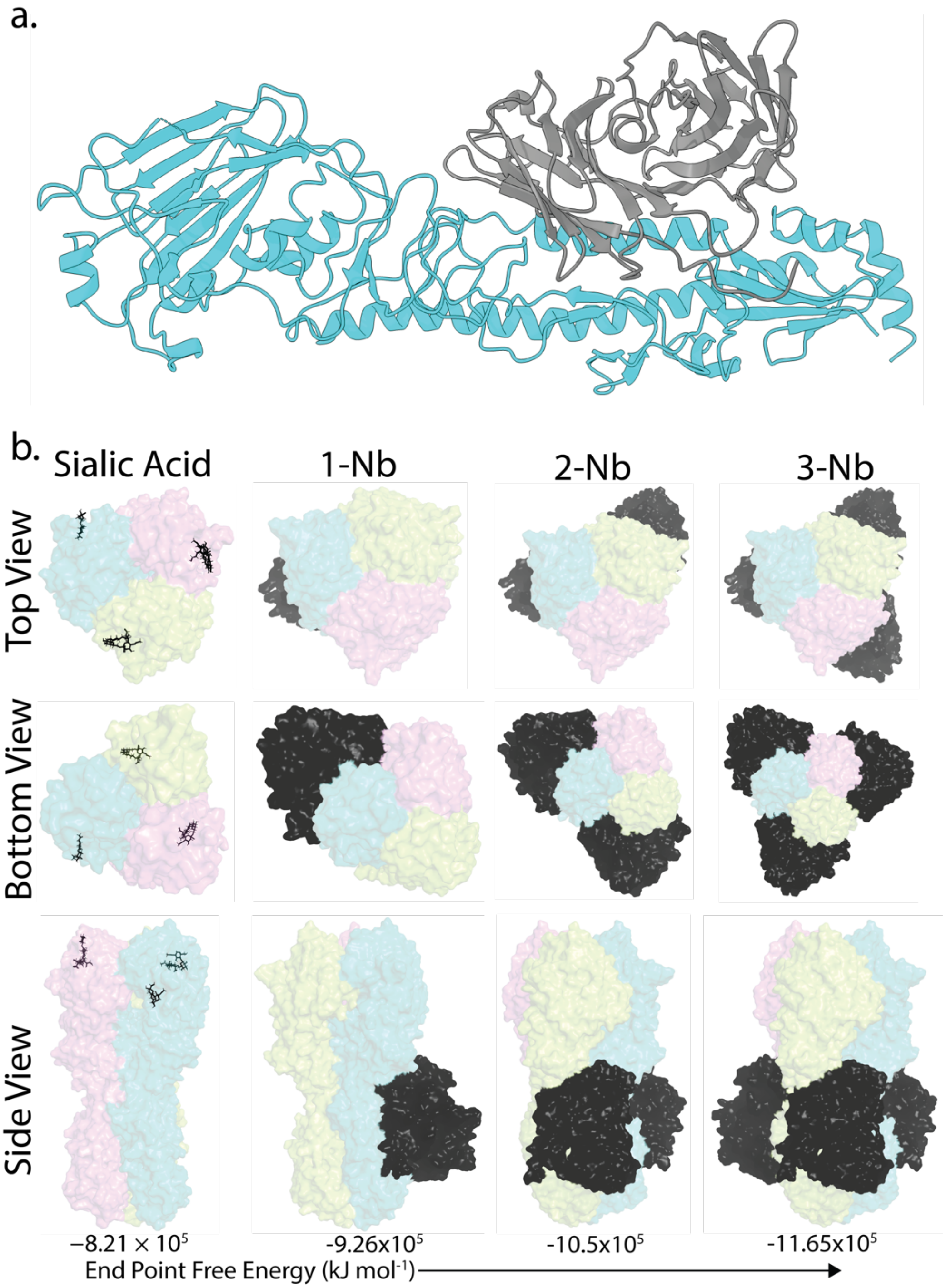

Figure S5. Computational studies of Sialic acid molecule and SD38-36 dual domain Nb with Influenza HA. (a) Molecular docking of HA monomer with ab initio modelled SD38-36 dual domain Nb reveals the stem region of HA as the primary binding target. (b) Using the HA-Nb monomer model, we generated HA trimer bound to one, two or three Nbs. Leftmost panels: black signal represents sialic-acid receptors. The 3-Nb complex mimics the exact geometrical patterning of HC-DDN. Energy minimization reveals that the 3-Nb design stabilizes the HA trimer significantly

higher than one or two Nbs or even the natural ligand- sialic acid. End point free energies are mentioned for each complex.

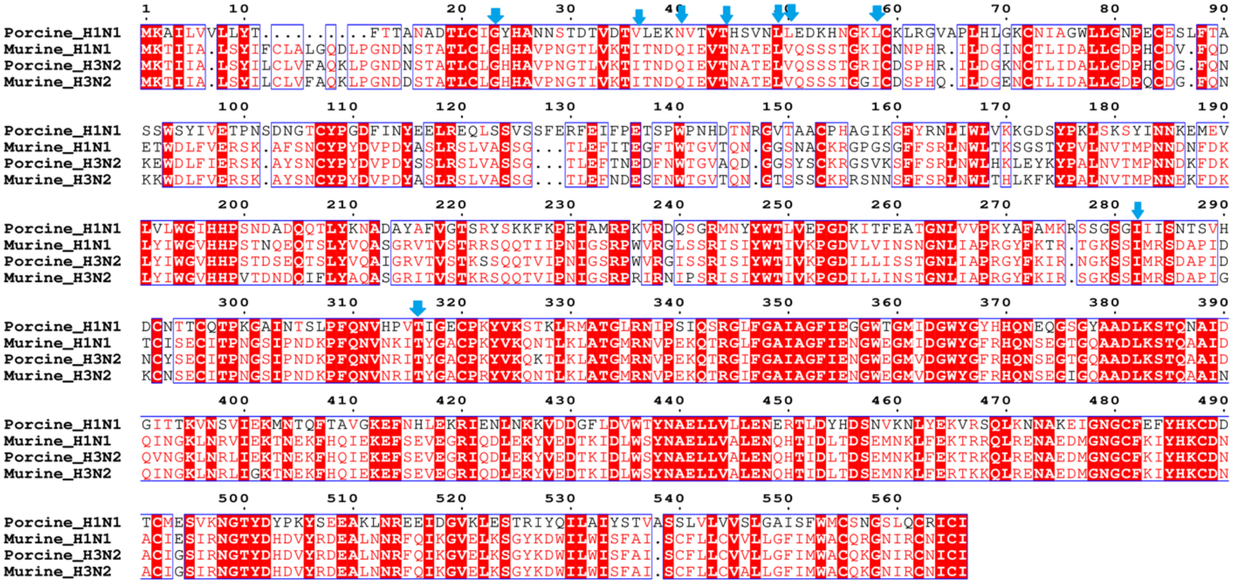

Figure S6. Multiple sequence alignment analysis (MSA) representation of HA from mice and pig adapted H1N1 and H3N2 strains, presented using ESPRIPT 3.0.

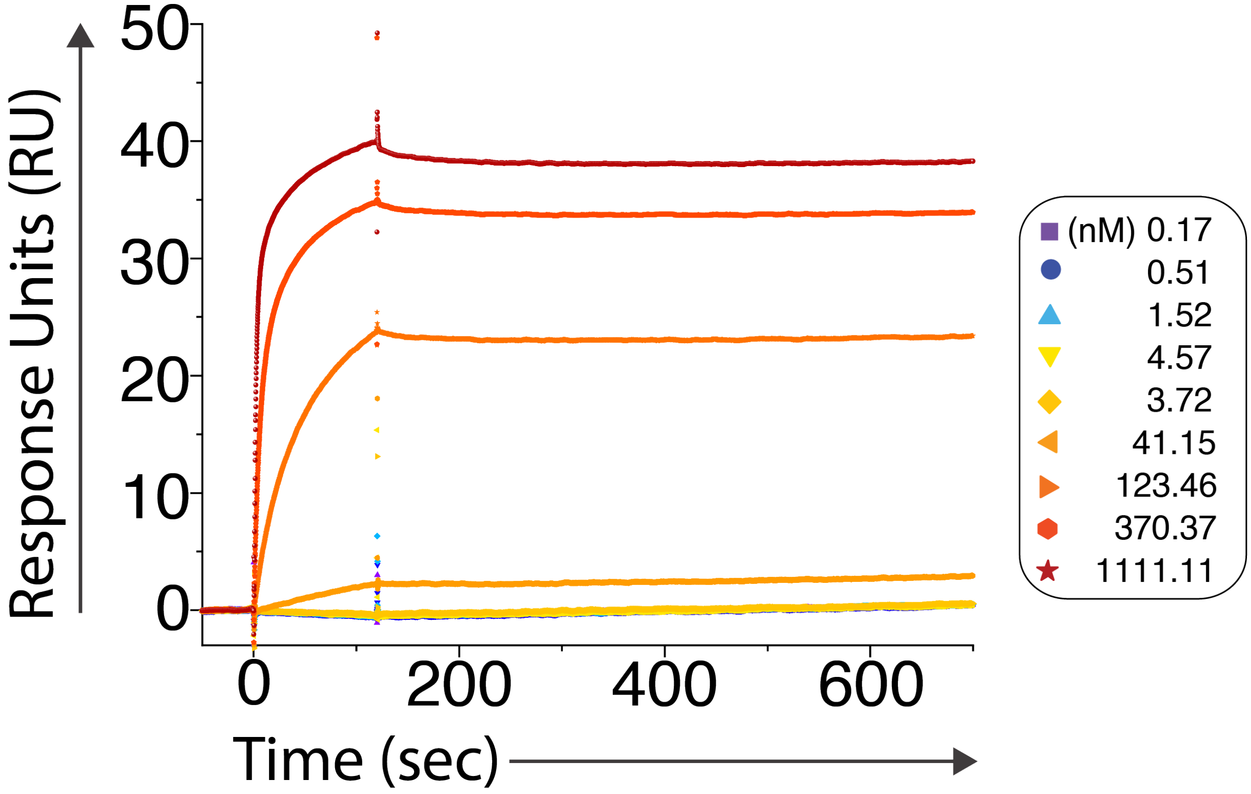

Figure S7. Surface plasmon resonance (SPR) analysis of ssDNA–nanobody (ssDNA–Nb) binding to H1N1 virions. Representative SPR sensorgram shows the binding interactions of free ssDNA–Nb conjugates with immobilized H1N1 virions. ssDNA–Nb was injected over the sensor surface for a 120 s association phase followed by a 600 s dissociation phase, using analyte concentrations ranging from 0.17 nM to 1.11 μM.

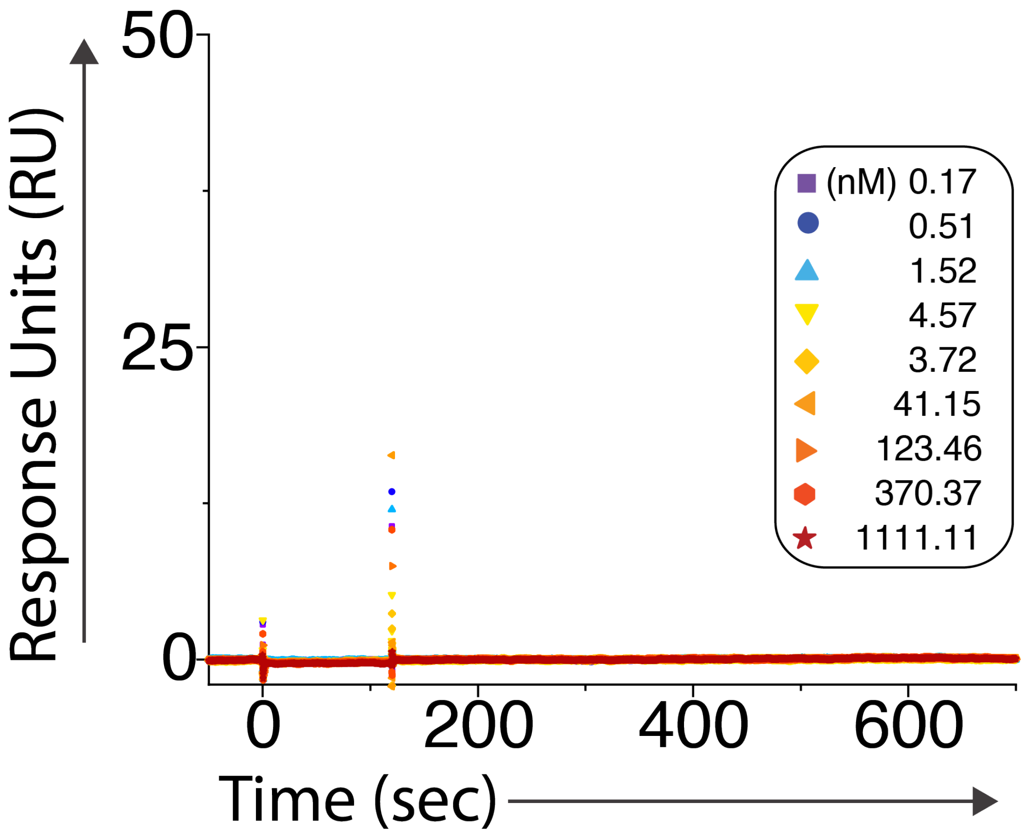

Figure S8. Specificity assessment of ssDNA–nanobody (ssDNA–Nb) conjugate binding using SPR. Representative SPR sensorgram shows the interaction profile of free ssDNA–Nb conjugates with immobilized SARS-CoV-2 virions. The ssDNA–Nb was flowed over the sensor surface with an association phase of 120 s and a dissociation phase of 600 s, across concentrations from 0.17 nM to 1.11 μM, confirming minimal non-specific binding to non-target virus.

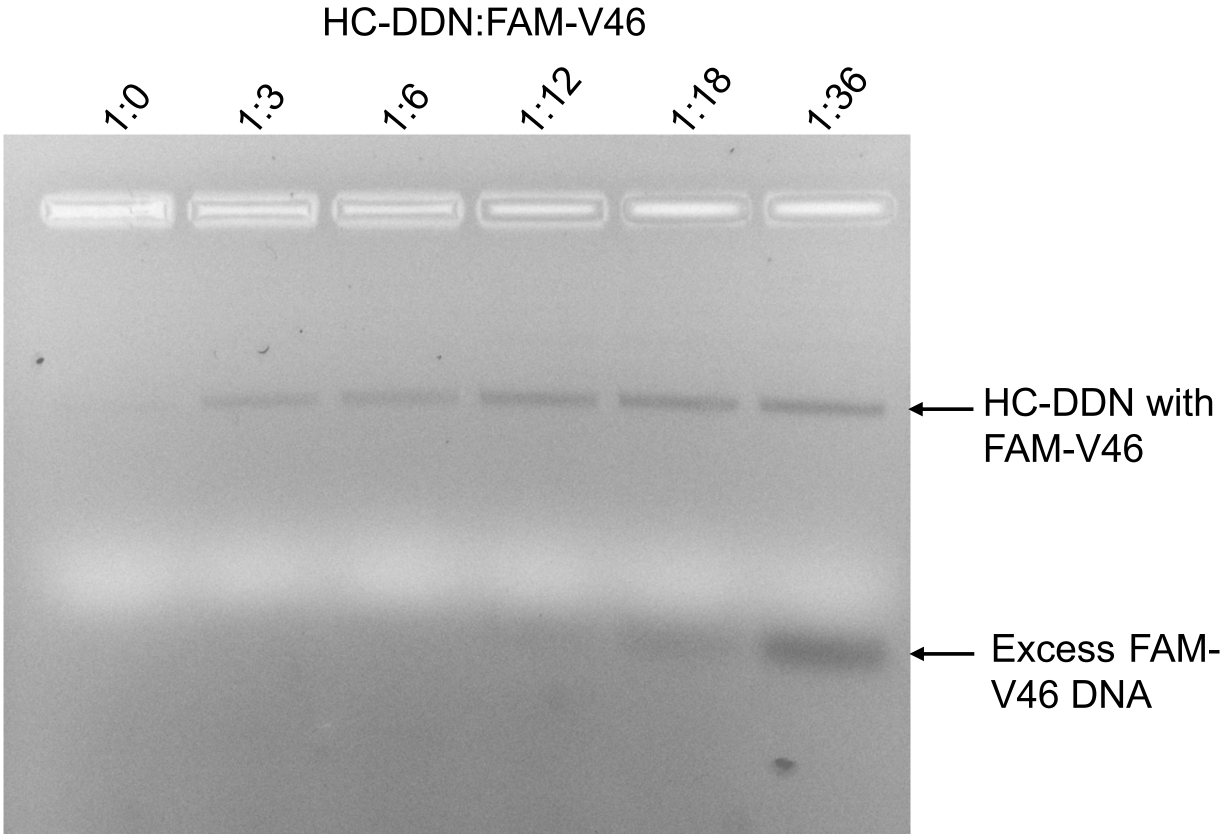

Figure S9. Verification of multivalent aptamer display on HC-DDN by agarose gel electrophoresis. HC-DDN (18 aptamer loading sites) was annealed with FAM-labeled V46 aptamers at scaffold-to-aptamer molar ratios of 1:0, 1:3, 1:6, 1:12, 1:18, and 1:36, followed by their characterization using a 1% agarose gel electrophoresis. Fluorescence imaging under the FAM channel showed progressively increased FAM fluorescence signal of the HC-DDN band with increasing loading ratios. Excess unbound aptamers appeared as a lower band at higher ratios. These results confirm successful incorporation of multiple aptamers to the docking sites displayed on HC-DDN.

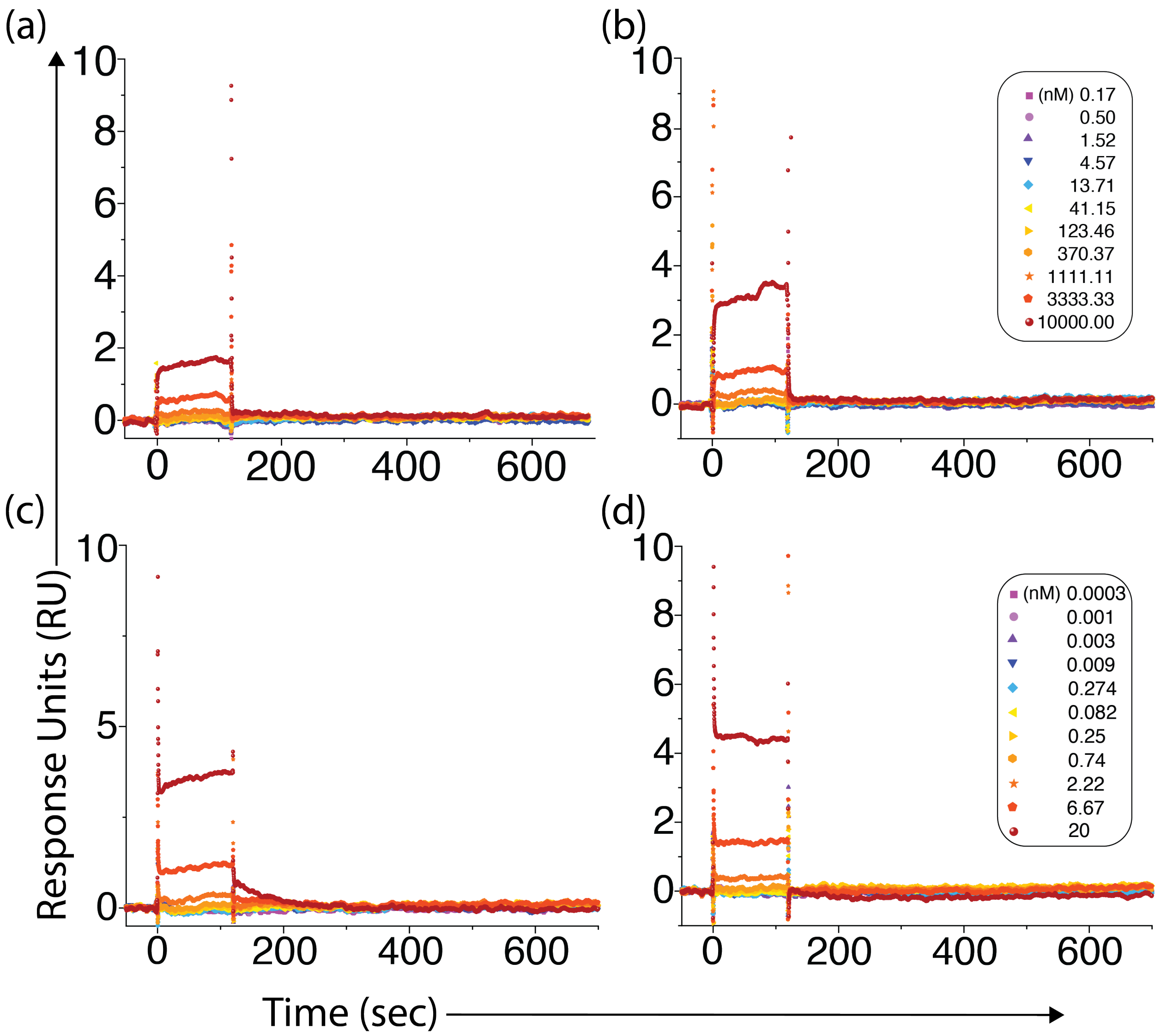

Figure S10. SPR-based binding analysis of UHA-2 aptamer constructs. (a–b) SPR sensorgrams illustrating the interaction of the free UHA-2 aptamer with immobilized H1N1 (a) and H3N2 (b) virions, tested across a concentration range of 0.17 nM to 10 µM. (c–d) Corresponding SPR profiles showing the binding of the HC–Apt (or HC-UHA2) constructs to H1N1 (c) and H3N2 (d) virions, recorded at concentrations from 3 pM to 20 nM.

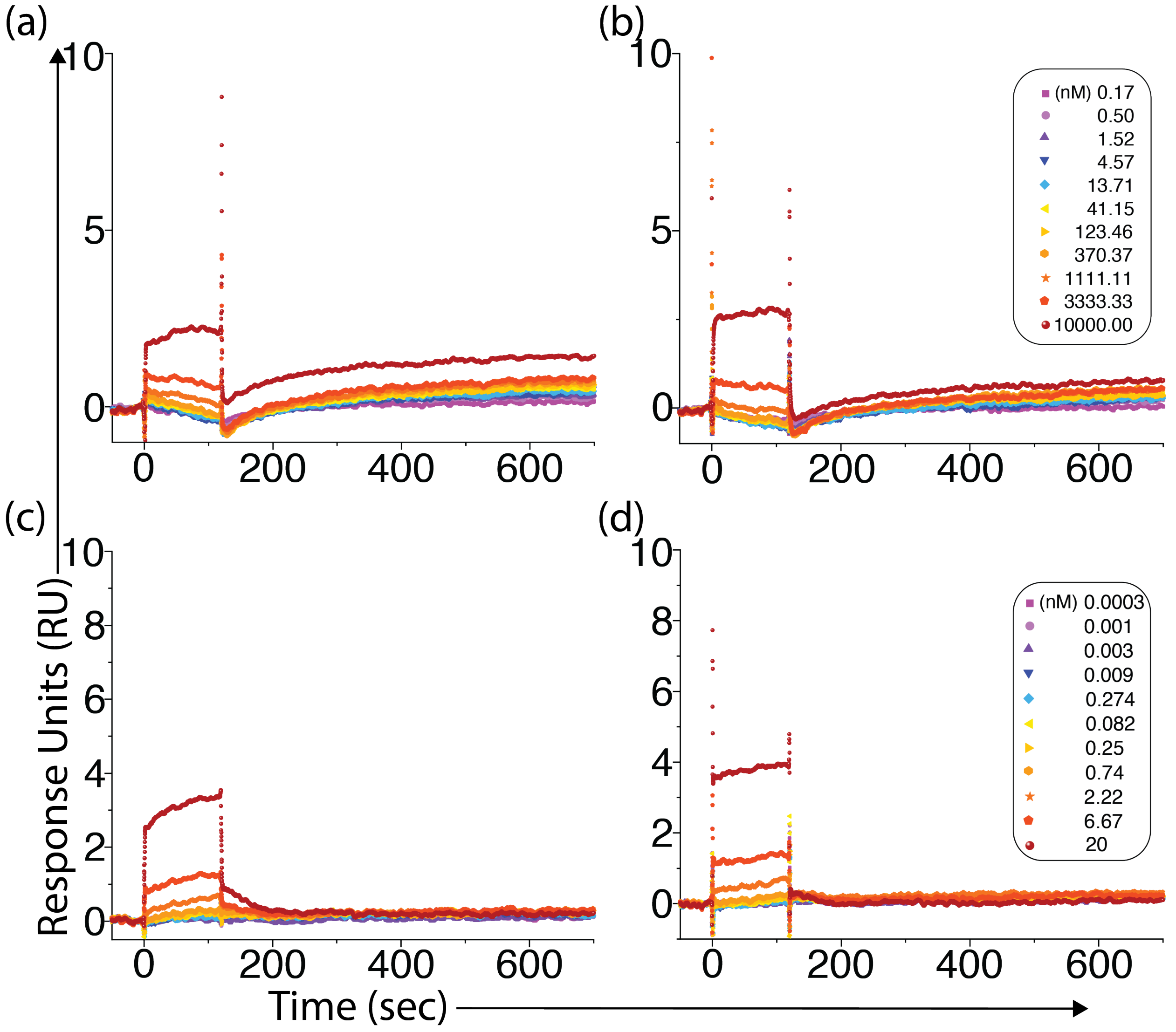

Figure S11. SPR characterization of V46 aptamer-derived binders. (a–b) Representative SPR sensorgrams displaying the binding response of the free V46 aptamer to immobilized H1N1 (a) and H3N2 (b) virions over a concentration range of 0.17 nM to 10 µM. (c–d) SPR sensorgrams showing how the HC–V46 constructs interact with H1N1 (c) and H3N2 (d) virions, tested at concentrations between 3 pM and 20 nM.

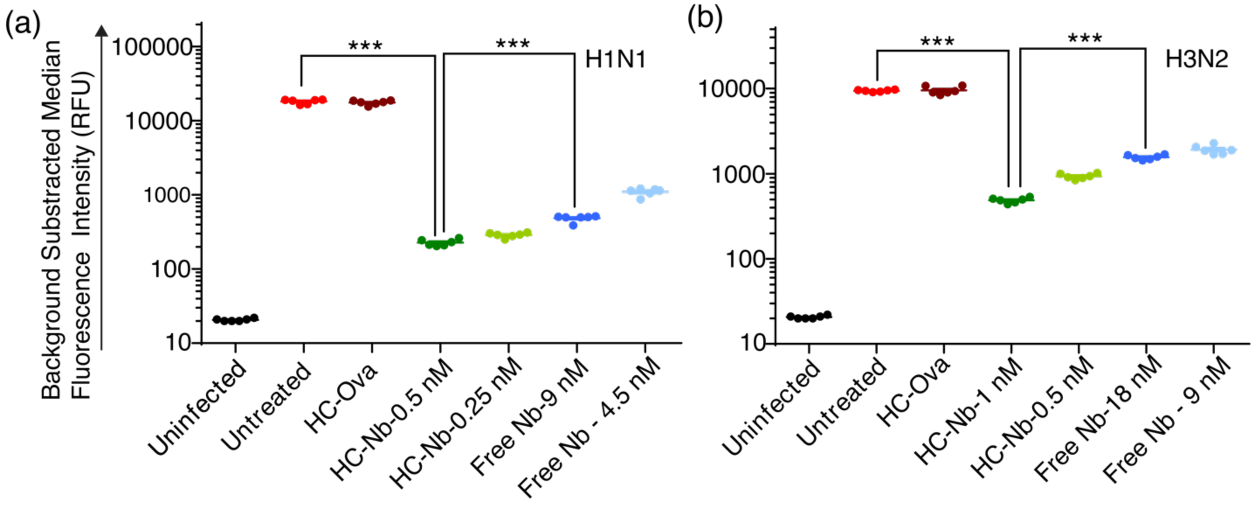

Figure S12. Quantitative flow cytometry analysis of viral load at 1 hour post-infection for murine-adapted IAV treated with HC–Nb and free nanobodies. Flow cytometry–based quantification of intracellular viral load for mouse-adapted influenza A virus: (a) H1N1 and (b) H3N2 pre-treated with the HC–Nb construct, free nanobodies, or control groups (uninfected, untreated virus, and HC–Ova) at varying concentrations.

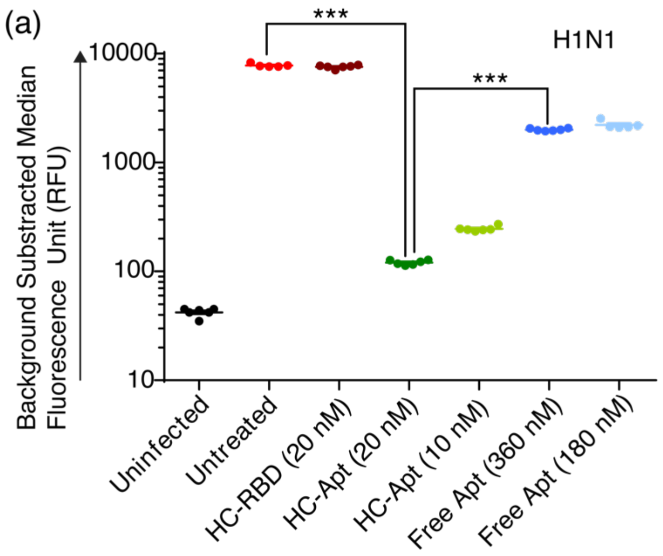

Figure S13. Flow cytometry measurement of viral burden at 1 hour post-infection for murine-adapted IAV treated with HC–Apt, and free UHA2 aptamers. Flow cytometry–based quantification of intracellular viral load for mouse-adapted influenza A virus: (a) H1N1 and (b) H3N2 pre-treated with the HC–Apt construct, free UHA2 aptamers, or control conditions (uninfected, untreated virus, and HC–RBD) across multiple concentrations. *****$p < 0.0001$.

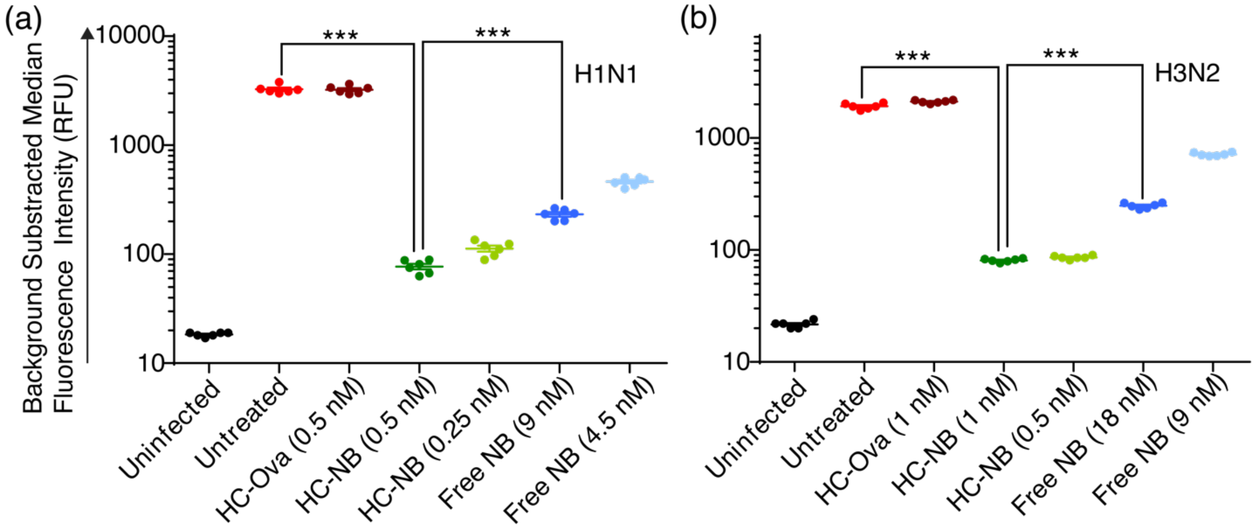

Figure S14. Flow cytometry–based quantification of viral load at 1 hour post-infection for swine-adapted IAV treated with HC–Nb, and free nanobodies. Flow cytometry–based quantification of intracellular viral load for swine-adapted influenza A virus: (a) H1N1 and (b) H3N2 pre-treated with the HC–Nb construct, free nanobodies, or control groups (uninfected, untreated virus, and HC–Ova) at varying concentrations.

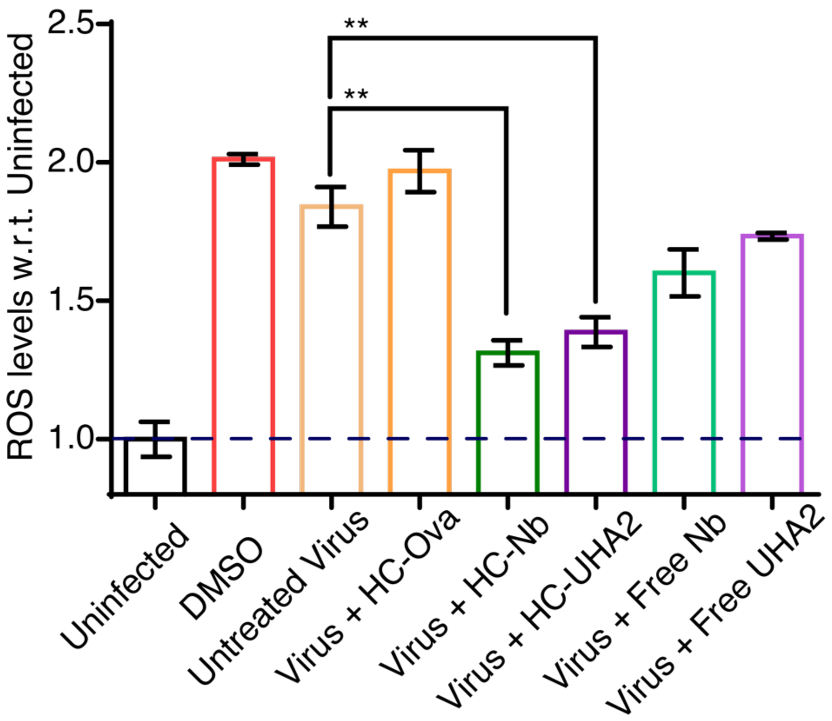

Figure S15. Pretreatment of H1N1 virus with HC-Nb, and HC-UHA2 significantly reduce ROS production in cell post infection. 10% DMSO acts as a positive control for ROS induction, uninfected cells serve as background control, n=4, Bars represent mean and SEM; **p < 0.001.

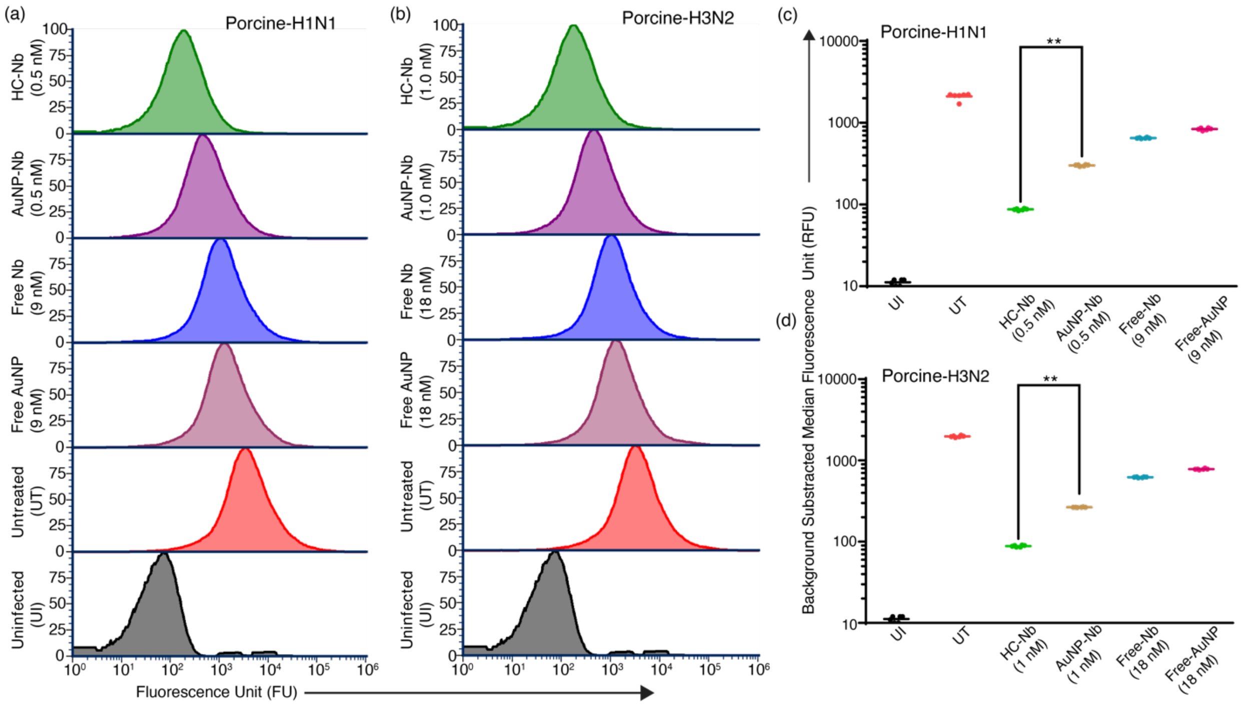

Figure S16. HC-DDN construct provides higher antiviral efficacy compared to gold nanoparticle (AuNP) against porcine-adapted influenza A viruses (IAV) using nanobody (Nb). (a) Flow cytometry shows that HC–Nb blocks H1N1 entry into porcine epithelial cells more efficiently than an equivalent dose of AuNP–Nb 1 h post-infection. (b) A similar effect is observed for the H3N2 subtype, highlighting cross-subtype efficacy of HC-Nb construct. Controls include untreated virions and uninfected cells. Flow cytometry–based quantification of intracellular viral load for (c) H1N1 and (d) H3N2 pretreated with HC–Nb, AuNP–Nb, free Nb/AuNP, or controls (uninfected, untreated virus) across multiple concentrations. Bars represent mean; $^{**}p < 0.001$.

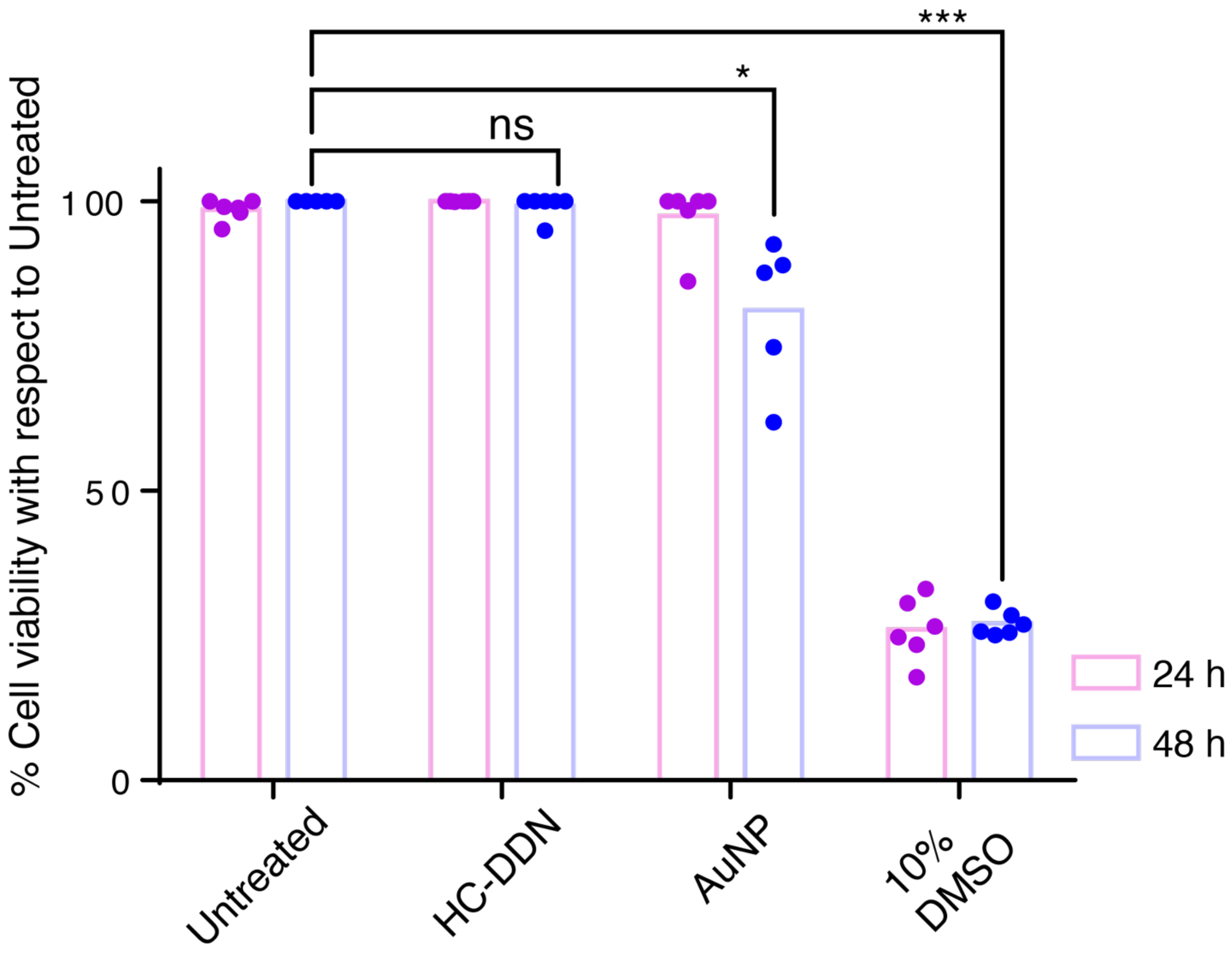

Figure S17. Gold nanoparticles (AuNP) affect cell viability. MTT assays performed 48h post treatment shows that AuNP reduces cell viability by approximately 25%, limiting its use as a probable therapeutic carrier. HC-DDN, however, has no effect on cell viability. DMSO-treated uninfected cells served as a positive control for cell death. Bars represent mean; *p < 0.01, ***p < 0.0001.

Table S1. Comparison of Generic Multivalent Platforms with HC-DDN Programmed Presentation (Valency, Spacing, Orientation)

**Design axis**	**Generic multivalent coverage (e.g., MATCH[9] / DNA-Star[10] / SNAP archetypes[11])**	**HC-DDN programmed presentation (this work)**
Scaffold & size	DNA Nanoscaffolds/NPs optimized for local targets: MATCH ~5.8 nm tetrahedron; DNA-Star ~42 nm star; SNAP ~5 nm AuNP core.	Honeycomb DNA origami ~124 nm, dimensioned to approximate influenza virion (~130 nm).
Target fit	MATCH: single SARS-CoV-2 spike trimer (three RBDs). DNA-Star: DENV sensing (assumes relatively immobile epitopes). SNAP: general NP-aptamer display.	Influenza virion-scale engagement: HA trimers (~16 nm length; ~9 nm head) distributed heterogeneously with inter-trimer spacings of several–tens of nm.
Valency control	Variable/heterogeneous: Au–thiol conjugation (SNAP) yields mixed ligand numbers; DNA nanoscaffold cap valency at low counts.	Fixed, site-defined valency via addressable staple positions; stoichiometry set by design rules.
Spacing control	Limited or implicit; nanoscaffold can't bridge multiple HA trimers at virion scale; inter-ligand spacing often stochastic (especially on AuNPs).	Nanometer-precise inter-ligand spacing (programmed node-to-node distances) to match HA trimer separations and enable multi-trimer engagement.
Orientational control	Mixed orientations common (especially with Au–thiol); optimal binder presentation fraction reduced.	Programmed orientation via DNA strand polarity and defined attachment geometry increases fraction of optimally presented binders.
Multivalency "scale"	Primarily single scale: local avidity (e.g., one trimer).	Dual-scale: (i) trimer-matched local clusters for avidity and (ii) virion-scale coverage to engage many HA trimers simultaneously.
Binder class	Aptamers only	Direct head-to-head on same scaffold: nanobody-decorated HC-DDN vs aptamer-decorated, with nanobodies outperforming in cytoprotection.
Mechanistic outcome	Local blocking/sensing; limited ability to neutralize intact, mobile, membrane-embedded epitope fields.	Cross-engagement across the virion, reducing entry across H1N1 and H3N2.
Reproducibility & interpretability	Stochastic ligand numbers/orientations complicate avidity analysis and reproducibility (notably with AuNPs).	Deterministic layouts(visualizable/simulatable), transparent mapping from design to function.

Scaffold effects & safety	AuNP cores can accrue protein corona, alter uptake/immune pathways; cytotoxicity seen at low–mid doses.	DNA-only scaffold avoids inorganic core effects; no detectable viability loss in MTT at 48 h.
General limitations	Geometry mismatches for large, mobile epitope fields; heterogeneous conjugation chemistry.	Larger build complexity but purpose-built for influenza geometry; supports rigorous controls and scaling.

Table S2. Oligonucleotide Sequences Used in HC-DDN Assembly.

Sequence Name	Sequence
strand_1	ACG GAA CAT TTT TCA TTA TTA CAA AAA CGA AAG ATT TTT TTG CAA AAG A
strand_2	TCG TGG GAG AGG CGG TTT TTT TTT GCG TAT TGG GCG CTG GTT CCG
strand_3	TCG CGT TTG ACG GGG AAA GCC GGC TTT TTT GAA CGT GG
strand_4	ATT AGC TGG TTT GCC TTT TTT CAG CAG GCG AAA AAT CCC TTA TTT TTT TAA ATC AAA
strand_5	GTC TTT ACT TTT TCT GAC TAT TAA TTA AGA GGA ATT TTT TCC CGA AAG ACG CGA A
strand_6	GTA CTT TCC TCG TTA TTT TTT TAA TCA GAG CGG GAG CGG ATT GCA
strand_7	CGC AAT AGC AGC CGT ATC AGG GCG ATT TTT GGC CC
strand_8	GCC GCG CTT AAT GTT TGG ATT ATA CTT CTG AAC ACC ACA CCC
strand_9	GCT GGC AAG TGG TAA CAG TAC CTT TTA CAT CGG CGC TAG GGC
strand_10	CGC AAT AGC AGC AAA TCG GAA TCG GCC AAC GCG CGG CCA GCT GCA TTA ATG AAC CCT AA
strand_11	CAA CGT TAC AAA ATC TTT TTT TCG CAG AGG CGA ATT ATG CGT AGA
strand_12	TTG AAT ACC AAG TCA AAG GGC GAA AAA CCG TCC CTG ATT GCT
strand_13	AAG AGT CCA CTC AAA ATT AAT TAC ATT TAA CAA GTT TGG AAC
strand_14	AAG CAT AAA GTA CCG TTT TTT TCA AAA GGT AAA GTA AAA AGC CAA
strand_15	AAT AAG AGA ATC GTT TTT ATT TTC ATC GTA GGT CGA GCC AGT
strand_16	CGC AAT AGC AGC ATA CAC TTA GTT AGC GTA ACG ATC CAC AGA CAG CCC TCA AAA ACA CT
strand_17	CAA CGC CAA CAT TGA AAT ACC GAC CGT GTG ATC CAT ATT TAA

strand_18	TTA CTA GAA AAA GTT TAG TAT CAT ATG CGT TAT ACA GTA GGG CTT ATT TTT TTT TGA GA
strand_19	TTG CAC GCT AAC GAG TTT TTT TGT CTT TCC AGA GCC TAT TAA ACC
strand_20	ATG CGC TAT TTT TGA ATG GCT ATA TTT ACA TTG GCA GAT TTT TTT TCA CCA GTC AGA AG
strand_21	GAA GCC TTA AAG TAG TAG CAT TAG CCG GAA GCG GGA GGT TTT
strand_22	ACG CGA GGC GTG ACG GGC AAC AGC TGA TTG CCT ATT CTA AGA
strand_23	AAG CGG TCC ACA TTT TCC CTT AGA ATC CTT GAG AGT TGC AGC
strand_24	TCA ATA GTG AAT TTA TTT TTT TCA AAA TCA TAG GTC TTG AAT AAC
strand_25	AGA AGA GTC AAA TAT ATT TTA GTT AAT TTC ATT AAG ACG CTG
strand_26	ATC GAA ATA AGG CGT TTT TTT TAA ATA AGA ATA AAC CCA ATC GC
strand_27	CTT TAA ACA GTG GTC ATT TTT GCG GAT GGC TTC CCT CAA ATG
strand_28	CGC AAT AGC AGC CTA TCG GCT TTT TTT GCT GGT AAA CAG GAA AAA CTT TTT TTC TCA TG
strand_29	AGA GAG TAC CTT TTT TGC TCC TTT TGA TAA GAT CAG AAA ACG ATT TTT TTA ATG ACC A
strand_30	ACA AGA ACT GAT AGC TTT TTT TCT AAA ACA TCG CCA TCA AAA GAA
strand_31	ACC TGA AAG CGT CAA ATA TCA AAC CCT CAA TCA ACC CTT CTG
strand_32	TAA TAA AAG GGA GAT AAT ACA TTT GAG GAT TTA CAC GAC CAG
strand_33	TTG TAT CCT TTG CCC TTT TTT TAA CGT TAT TAA TTT TTT AGG AGC
strand_34	ACT CGT ATT AAA GCA ATA CTT CTT TGA TTA GTC AAT TCG ACA
strand_35	AAG AGT CTG TCG CGG AAT TAT CAT CAT ATT CCC ACC GAG TAA
strand_36	CGC AAT AGC AGC TAT AGA AGT TTT TTG CTT ATC CGG CTT CA
strand_37	ATT CCG CAA AGA CAC TTT TTT TAC GGA ATA AGT TTA TAC GGA ATA
strand_38	TAT AAA AGA AAA TTA AAG GTG AAT TAT CAC CGG GTG GCA ACA
strand_39	TTA CGA GCC CCA CAA GAA TTG AGT TTT TTA AGC CC
strand_40	TAT GTT AGC AAA TAA GAG CAA GAA ACA ATG AAT ATT ACG CAG
strand_41	GTA AGC AGA TAG CTA AAG TTA CCA GAA GGA AAC CGA ACT GGC ATG ATT TTT TTT AAG AC
strand_42	GCC TAT TTT CGG TCA TTT TTT TAG CCC CCT TAT TAG CAT TGA GGG
strand_43	CGC AAT AGC AGC TTA TGC GAA ATC AAC GTA ACA AAC GGA TAT TCA TTA CCC ATT TTA AG
strand_44	CCG TAA TCA GTC TTG ATA CCG ATA GTT GCG CCG ATA GCA GCA

strand_45	AGC AAG GCC GGA GCG CAT TAG ACG GGA GAA TTC ATT ACC ATT
strand_46	GCC TTT ACA GAC TGT TTA GCT ATA TTT TCA TTG AAA ATA GCA
strand_47	CGC AAT AGC AGC AAT AAC GTA GAA AAT TTT TTT TCA TAC
strand_48	TCC TAT AGC AAT AGC TTT TTT TAT CTT ACC GAA GCC AAC AAT AG
strand_49	TAC TGG CAG GTC AGA TTT TTT TGA TTG GCC TTG ATA TTC AGA GCC
strand_50	CAG GAG GTT GAG GTA ATA AGT TTT AAC GGG GTC CAG CAT TGA
strand_51	CTG AGG CCA CGC ATA ACC GAT ATA AAG GCT CCA AAA GGA TTT TTC CTT TAA TTG AGA AC
strand_52	GAG CCG CCA CCT ATC GGT TTA TCA GCT TGC TTA CCA CCC TCA
strand_53	TTC ATA ATC AAT TTA CCG GAA CCA GAG CCA CCC AGA ACC GCC ATT TTT TTC CTC AG
strand_54	TAT ACC TGC CTA TTT TTT TTT TGG AAC CTA TTA TTC TAG CGT CAT
strand_55	AGT TAA TGC CCA GTA TAG CCC GGA ATA GGT GTC CGT ATA AAC
strand_56	CAG TGC CAC CCT CAG TTT TTT TAC CGC CAC CCT CAG AGT ACC AGG
strand_57	CCC TCA GAA CCT TCA GCG GAG TGA GAA TAG AAA GTA CCG CCA
strand_58	GGA AAC TAA AGA CTT TTT TTT TTT CAT GAG GAA GTT TTT TTC TGT
strand_59	CGC AAT AGC AGC CAC CAC TTT TTT TAG AGC CGC CGC AGT G
strand_60	CGT CAC CCT CAA ATT CTG CGA ACG AGT AGA TTT TTG CGG GAT
strand_61	CGC AAT AGC AGC TAA TAA ACA TAA CCC TCG TTT ACG TAA GAG CAA CAC TAT ACG AAC TA
strand_62	GGC ACC AAC CTG GTA GAA AGA TTC ATC AGT TGC CAC TAC GAA
strand_63	ATG GGA TTG ACG TTA GTA AAT GAA CCA TTA AAC GGG TAA ATT TTT TAT ACG T
strand_64	GGC TAG TAC AAC GGA TTT TTT TAT TTG TAT CAT CGC CGT TTC GTC
strand_65	GCG CGA AAC AAT GAG ATG GTT TAA TTT CAA CTA TTA TAC CAA
strand_66	CGA GAA ACA CCA GGC GCA GAC GGT CAA TCA TAC TTG CCC TGA
strand_67	CAT CTT TGT TTT TTA CCC CCA GCG TTA ATC ATT GTT TTT TTG AAT TAC C
strand_68	GCA GAC ACC GCC TGC TTT TTT TAC AGT GCC ACG CTG AGC TGG CTG
strand_69	ACA GAG GTG ACG TTG GGA AGA AAA TTT TTT ATC TAC GT
strand_70	GAT ACA TAA CGT TTA AAT ATG CAA CTA AAG TAA ACT AAT GCA

strand_71	AAA GCC TGG TTT CCT AAT GAG TGA GCT AAC TTC CAG TCG GGT TTT TTA ACC TG
strand_72	CGC AAT AGC AGC CGA GAA AAG CAC GTA TAA CGT GCT ATG GTT GCT TTG ACG GGA AGG GA
strand_73	CGC AAT AGC AGC AGT CAG ATC CAA ATA AGA AAC GAA TAT TAT TTA TCC CAA GGG TAA TT
strand_74	AGA ATA GAT CAA GTT TTT TGG GGG TGA ACC ATC ACC CAA CCC GAG AT
strand_75	CCA GAC TTT TTT TGG AAG CAA ACT CCA AGC GCT CAC TGC CCG CTT CAC ATT A
strand_76	ATT GCG TTC AGG TCA GGA TT
strand_77	AGG GAG CCT TTT TTC CCG ATT TAG AGC TTT AAT TCG AGC TTC AAA TTC AAA TA
strand_78	GAT GAT GGC TTT CAT CAA TAT AAT CCT GAT TGC GCC GCT ACT TTT TTG GGC GC
strand_79	TTA GAA CCT ATT CAA AAT TAT TTG CAC GTA AAT TTA ACG TCA GTT TTT TTG AAT A
strand_80	TTT TCA GGA CAG AAA TAA AGA AAT TTC ATT TCA ATT A
strand_81	CCT GAG CAA AAG ATA TGA AAC AAA CAT CAA GAA AAA TTA AAG AAC GTT TTT TGG ACT C
strand_82	AGG GTT GAT TTT TTG TGT TGT TCC ATT TCA TTT
strand_83	CGC TCA ACA AAT TCT TAC CAG TAT TTC TGT CCA GAC
strand_84	TCC CAT AAT CGG CTG TTT TTT CTT TCC TTA TCA CTC ATC GAG TTT TTT ACA AGC
strand_85	AGC TGA AAA TTT GCA TCA ATT CTA CTA ATA TCA AGA TTA GTT TTT TTG CTA TT
strand_86	TAG CAA GCA TCC TGA ATC TTA CCA ACC CAG CTA CAA TTT TAA ATC AGA
strand_87	GTG ATT TAG CGA ACC TTT TTT TCC CGA CTT GCA TAA AGT GT
strand_88	AAA TCG GCA AAT CCT GTT TGA TGG CAG GGT GGT TTT TCT TTT TTT TCA CCA
strand_89	GAA TTA CCT TTT TAA TGG AAA CAG TAC ATA TGT AAA TCG TCT TTT TTC TAT TA

strand_90	AGA AAG CGT TTT TTA AAG GAG CGG GGA GAA ACA ATT TTT TTA ACG GAT T
strand_91	CTT GCT TCA ATC AAT ATA TGT GAG GAG AGA CTA CCT T
strand_92	TTT AAC CTC CGG CTT TGG GTT ATA TAA CTA TAT GTG AAC GCG AGA ATT TTT TAC TTT T
strand_93	AAG ACA AAA AAT GCT GAT GCA AAT ACC GGA ATC ATA A
strand_94	ATG TGT CAT AAA TAT TTT TTT TCA TTG AAT CCA GAG CTT AA
strand_95	CAT ACA GAC GAC GAT TTT TTT AAA ACC AAA AAG AGG GGG TAA TTT TTT AGT AAA
strand_96	GAA ATA CCT CCA ATA CTG CGG AAT CTT AGA CTG GAT AGC GTA CAT TTT G
strand_97	TAA ATC AAC CGC CAG CCA TTG CAT ATC CAG AAC AAT ATT AAA ATC AG
strand_98	AAT GAA AAA TCT TTG CAT CAC CTT GCT GAA CCT AAG AAT ACG TTT TTT TGC ACA G
strand_99	GAG CGC TAT TTT TTA TAT CAG AGA GAT AAA TGT AGA AAC CAA TCA ATC CTA AT
strand_100	AGT TGG CAA ATA GTT GAA AGG AAT TGA GGA AGC TAA TAG ATT ATT TTT TAG CCG T
strand_101	CGC AAT AGC AGC ACG CTC AAT TTT TTT CGT CTG AAA TGG ATT TAG TCT TTA
strand_102	ACT AAC AAG TTA TCT AAA ATA TCT AAA AGT TTG AGT A
strand_103	ACA TTA TCA TTT TTA CAA AGA AAC CAC CAG AAG GAC ATC ACG CAA ATT TTT TTA ACC G
strand_104	ATT TGT GTT TTT ATA TTT TTT TTC AGT GAG GCT GAT TAT CA
strand_105	TCA AAA AGT AGT CAG AAG CAA AGC TAA ACA GGA GGC CGA TTT TTT AAA GGG
strand_106	GTA GAA GGC CAG AAT CCT GAG AAT AGA CAG GAA CGG TAC AAC TCA AA
strand_107	CCC AAA AGA GGA AAC GCA ATA ATA TTT GTC ACA ATC A
strand_108	ATA GAA AAT TCA TTT TAC CAG CGC CAA AGA CAA AAT AAA TAT TGA CTT TTT TGA AAT T
strand_109	AGG GAA GGG GGC GAC ATT CAA CCG GTT TGC CAT CTT

strand_110	AGC CTC GAG GTG AAT TTT TTT CTT AAA CAG AGC GAC AGA ATT TTT TTA AGT TT
strand_111	CCG CCT TTT TTT GGC CCT GAG AAA ACA
strand_112	CAT TAG ATA TTT TCG CAA ATG GTC AAT AAC GAG AAT AAC ATT TTT TTA AAA CA
strand_113	AGC CTT TTT TGT TTA TTT TTT TCG TCA AAA ATT GGG GCG CG
strand_114	AAG TAC CGC ATT CCA AGA ACG GGT AAT TTG CCA GTT ACA ATT TTT ATA AAC
strand_115	CGC AAT AGC AGC TTG GGA ATT GCG CGT TTT CAT CGG CTT AGC GTC AGA CTG TAA GAG CC
strand_116	GAC GAC AAT AAT TTC ATG TTC AGC TAA TGC AGT GAA CAA GAA ATT TTT TAT AAT A
strand_117	GAC CTA TTT TTA TTT AAT GGT TGT AA
strand_118	ATA AGT CCA ACG CGC CTG TTT ATC CTT TTT AAG AAA A
strand_119	GCC ACC CTA CCG GAA CCG CCT CCC TCA CAA ACA AAT A
strand_120	AAT CCT CAT TAA ATA ATG GAA AGC GCA GTC TCT GAT TTG ATG ATA CTT TTT TGG AGT G
strand_121	ACA TGG CTA TTT ACC GTT CCA GTA GAA ACA TGA AAG T
strand_122	ATT AAG AGG CTG ATC TCA AGA GAA GGA TTA GGA TTT GCC GTC GAG ATT TTT TGG TTG A
strand_123	CGC AAT AGC AGC CGT ACT TTT TTT CAG GAG GTT TAG GAA CAA CTA TTT TTT AAG GAA TT
strand_124	CGG ATA AGA GCG GGG TTT TGC TCA GCC ACC ACC CTC
strand_125	CAT TCT AAA GTT TTG TTT TTT CGT CTT TCC ATT GCT AAA CAA TTT TTT TTT CAA
strand_126	GAA GTT TCA TTT ATA TAA CAG TTG ATT CCC GCA GCG AAA GAT TTT TTA GCA TC
strand_127	GCG AAT ATA CAG AGG CTT TGA GGC GAG GGT AGC AAC GGC ATA ATT TT
strand_128	CGC CTT GCA GGG AGT TTT TTT TAA AGG CCG CTT AGT TTG AC
strand_129	GGG AAA ACG TCA CCA TTT TTT TTG AAA CCA TCG ACA ATG ACA ATT TTT AAC CAT

strand_130	CGC AAT AGC AGC TTC ACG TTT TTT TTG AAA ATC TCC AAA AAA TTC GGT CG
strand_131	ATT TTC AGG GAT TTA AGC CCA ATA GGA ACC CAA AAC TAC AAC GTT TTT TCT GTA G
strand_132	CGC AAT AGC AGC TAG CGA TTT TTT TAG CTT AGA TCT TCT
strand_133	ATA AAT CAC CGA CTT GTT TTT TAG CCA T
strand_134	ACC AGT ACT GTA CCG TAA CAC TGA TGA TAA ATT GTG T
strand_135	CGA AAT CCG CGA CTC CAT GTT ACT TAG CCG GAA CGA GAA CGA GTA GTT TTT TAA ATT G
strand_136	CTG ACC AAC TTA GAG GAC AGA TGA ACG GTG TAC AAG AGT AAT CTT TTT TTG ACA A
strand_137	CCT TGA TTT TTT GTA ACA GTG CAT CAC
strand_138	ACC TTC ATC AGA CCA GGC GCA TAG GAG CCA GCA GCA
strand_139	GTT TTG CCT AGC GAG AGG CTT TTG TAA AAA TAC CGA ACG ATT TTT CCA CCA
strand_140	AAC TGG CTT TTT TTC ATT ATA CCA GTC AGG AGG CGG TCA GTA TTA AAG ATA AA
strand_141	TTG CTG AAT TTT TGC TGT AGC TCA ACA TGT CCA AAA GGA ATT TTT TTA CGA GG
strand_142	AAT GAG ATT TAG GAA TTT TTT TAC CAC ATT CCG GTG TCT G
strand_143	TAC ATA GCG GTC ACG TTT TTT TTG CGC GTA ACT AAT GGA AGG G
strand_144	CAA TAC ATT CTG GCC TTT TTT TAC AGA GAT AGA ATA TCT GGT C
strand_145	GAA CGC TGC TCA TTC TTT TTT TGT GAA TAA GGA GGG AAC CGA A
strand_146	ACT ACT CGA GGT GCC GTT TTT TTA AAG CAC T
strand_147	CGC AAT AGC AGC TAT TAG TTT TTT ACT TTA CAA AAA TAA CAT CAC TTT TTT TTG CCT GA
strand_150	CGC AAT AGC AGC TTT AGG TTT TTT TAG AGG CAT TTA ATC ATT ACC GTT TTT TCG CCC AA
strand_151	AGC AAA ATC ATT TTT TCC AGT AGC ACA ACT GAA CAC CTT TTT TCT GAA CAA

References

1. Laursen, N. S.; Friesen, R. H. E.; Zhu, X.; Jongeneelen, M.; Blokland, S.; Vermond, J.; van Eijgen, A.; Tang, C.; van Diepen, H.; Obmolova, G.; van der Neut Kolfschoten, M.; Zuijdgeest, D.; Straetemans, R.; Hoffman, R. M. B.; Nieusma, T.; Pallesen, J.; Turner, H. L.; Bernard, S. M.; Ward, A. B.; Luo, J.; Poon, L. L. M.; Tretiakova, A. P.; Wilson, J. M.; Limberis, M. P.; Vogels, R.; Brandenburg, B.; Kolkman, J. A.; Wilson, I. A., Universal protection against influenza infection by a multidomain antibody to influenza hemagglutinin. *Science* **2018,** *362* (6414), 598-602.
2. Baek, M.; DiMaio, F.; Anishchenko, I.; Dauparas, J.; Ovchinnikov, S.; Lee, G. R.; Wang, J.; Cong, Q.; Kinch, L. N.; Schaeffer, R. D.; Millán, C.; Park, H.; Adams, C.; Glassman, C. R.; DeGiovanni, A.; Pereira, J. H.; Rodrigues, A. V.; van Dijk, A. A.; Ebrecht, A. C.; Opperman, D. J.; Sagmeister, T.; Buhlheller, C.; Pavkov-Keller, T.; Rathinaswamy, M. K.; Dalwadi, U.; Yip, C. K.; Burke, J. E.; Garcia, K. C.; Grishin, N. V.; Adams, P. D.; Read, R. J.; Baker, D., Accurate prediction of protein structures and interactions using a three-track neural network. *Science* **2021,** *373* (6557), 871-876.
3. Krieger, E.; Joo, K.; Lee, J.; Lee, J.; Raman, S.; Thompson, J.; Tyka, M.; Baker, D.; Karplus, K., Improving physical realism, stereochemistry, and side-chain accuracy in homology modeling: Four approaches that performed well in CASP8. *Proteins: Structure, Function, and Bioinformatics* **2009,** *77* (S9), 114-122.
4. Pettersen, E. F.; Goddard, T. D.; Huang, C. C.; Meng, E. C.; Couch, G. S.; Croll, T. I.; Morris, J. H.; Ferrin, T. E., UCSF ChimeraX: Structure visualization for researchers, educators, and developers. *Protein Science* **2020,** *30* (1), 70-82.
5. Wang, M.; Hao, M.-C.; Huangfu, Y.; Yang, K.-Z.; Zhang, X.-Q.; Zhang, Y.; Chen, J.; Zhang, Z.-L., A Universal Aptamer for Influenza A Viruses: Selection, Recognition, and Infection Inhibition. *ACS Pharmacology & Translational Science* **2023,** *7* (1), 249-258.
6. Bhardwaj, J.; Chaudhary, N.; Kim, H.; Jang, J., Subtyping of influenza A H1N1 virus using a label-free electrochemical biosensor based on the DNA aptamer targeting the stem region of HA protein. *Anal Chim Acta* **2019,** *1064*, 94-103.
7. Song, T.; Cooper, L.; Galván Achi, J.; Wang, X.; Dwivedy, A.; Rong, L.; Wang, X., Polyvalent Nanobody Structure Designed for Boosting SARS-CoV-2 Inhibition. *Journal of the American Chemical Society* **2024,** *146* (9), 5894-5900.
8. Zhou, L.; Xiong, Y.; Dwivedy, A.; Zheng, M.; Cooper, L.; Shepherd, S.; Song, T.; Hong, W.; Le, L. T. P.; Chen, X.; Umrao, S.; Rong, L.; Wang, T.; Cunningham, B. T.; Wang, X., Bioinspired designer DNA NanoGripper for virus sensing and potential inhibition. *Science Robotics* **2024,** *9* (96), eadi2084.
9. Wan, S.; Liu, S.; Sun, M.; Zhang, J.; Wei, X.; Song, T.; Li, Y.; Liu, X.; Chen, H.; Yang, C. J.; Song, Y., Spatial- and Valence-Matched Neutralizing DNA Nanostructure Blocks Wild-Type SARS-CoV-2 and Omicron Variant Infection. *Acs Nano* **2022,** *16* (9), 15310-15317.
10. Kwon, P. S.; Ren, S.; Kwon, S. J.; Kizer, M. E.; Kuo, L.; Xie, M.; Zhu, D.; Zhou, F.; Zhang, F.; Kim, D.; Fraser, K.; Kramer, L. D.; Seeman, N. C.; Dordick, J. S.; Linhardt, R. J.; Chao, J.; Wang, X., Designer DNA architecture offers precise and multivalent spatial pattern-recognition for viral sensing and inhibition. *Nat Chem* **2020,** *12* (1), 26-35.
11. Sun, M.; Wu, Z.; Zhang, J.; Chen, M.; Lu, Y.; Yang, C.; Song, Y., Spherical neutralizing aptamer suppresses SARS-CoV-2 Omicron escape. *Nano Today* **2022,** *44*.
